# Integrative genomic and transcriptomic analyses illuminate the ontology of HER2-low breast carcinomas

**DOI:** 10.1186/s13073-022-01104-z

**Published:** 2022-08-29

**Authors:** Enrico Berrino, Laura Annaratone, Sara Erika Bellomo, Giulio Ferrero, Amedeo Gagliardi, Alberto Bragoni, Dora Grassini, Simonetta Guarrera, Caterina Parlato, Laura Casorzo, Mara Panero, Ivana Sarotto, Silvia Giordano, Matteo Cereda, Filippo Montemurro, Riccardo Ponzone, Nicola Crosetto, Alessio Naccarati, Anna Sapino, Caterina Marchiò

**Affiliations:** 1 Candiolo Cancer Institute, FPO-IRCCS, Candiolo, TO Italy; 2 Department of Medical Sciences, University of Turin, Turin, Italy; 3 Department of Oncology, University of Turin, Turin, Italy; 4 Department of Clinical and Biological Sciences, University of Turin, Turin, Italy; 5 IIGM-Italian Institute for Genomic Medicine, c/o IRCCS, Candiolo, TO Italy; 6 Department of Biosciences, La Statale University, Milan, Italy; 7 Department of Microbiology, Tumor and Cell Biology, Karolinska Institutet, Stockholm, Sweden; 8 Science for Life Laboratory, Stockholm, Sweden

**Keywords:** Breast cancer, HER2, HER2-low, Heterogeneity, Classification, Mutation, Actionable alteration, Gene expression, Transcriptome, Stratification

## Abstract

**Background:**

The “HER2-low” nomenclature identifies breast carcinomas (BCs) displaying a HER2 score of 1+/2+ in immunohistochemistry and lacking *ERBB2* amplification. Whether HER2-low BCs (HLBCs) constitute a distinct entity is debated.

**Methods:**

We performed DNA and RNA high-throughput analysis on 99 HLBC samples (*n* = 34 cases with HER2 score 1+/HLBC-1, *n* = 15 cases with HER2 score 2+ and *ERBB2* not amplified/HLBC-2N, and *n* = 50 cases with score 2+ and *ERBB2* copy number in the equivocal range/HLBC-2E). We compared the mutation rates with data from 1317 samples in the Memorial Sloan-Kettering Cancer Center (MSKCC) BC cohort and gene expression data with those from an internal cohort of HER2-negative and HER2-positive BCs.

**Results:**

The most represented mutations affected *PIK3CA* (31/99, 31%), *GATA3* (18/99, 18%), *TP53* (17/99, 17%), and *ERBB2* (8/99, 8%, private to HLBC-2E). Tumor mutational burden was significantly higher in HLBC-1 compared to HLBC-2E/N (*P* = 0.04). Comparison of mutation spectra revealed that HLBCs were different from both HER2-negative and HER2-positive BCs, with HLBC-1 resembling more HER2-negative tumors and HLBC-2 mutationally related to HER2-addicted tumors. Potentially actionable alterations (annotated by using OncoKB/ESCAT classes) affected 52 patients. Intra-group gene expression revealed overlapping features between HLBC-1 and control HER2-negative BCs, whereas the HLBC-2E tumors showed the highest diversity overall. The RNA-based class discovery analysis unveiled four subsets of tumors with (i) lymphocyte activation, (ii) unique enrichment in HER2-related features, (iii) stromal remodeling alterations, and (iv) actionability of *PIK3CA* mutations (LAURA classification).

**Conclusions:**

HLBCs harbor distinct genomic features when compared with HER2-positive and HER2-negative BCs; however, differences across IHC classes were also unveiled thus dissecting the full picture of heterogeneity across HER2-low disease. The HLBC-2E category harbors most distinctive features, whereas HLBC-1 seems superimposable to HER2-negative disease. Further studies are needed to ascertain whether the four genomic-driver classes of the LAURA classification hold prognostic and/or predictive implications.

**Supplementary Information:**

The online version contains supplementary material available at 10.1186/s13073-022-01104-z.

## Background

HER2 overexpression in breast cancer (BC) is strictly connected to the underlying presence of *ERBB2* amplification [1] to which they become addicted [2], providing the basis of successful treatment of these tumors with traditional and novel anti-HER2 agents [1]. BCs have been historically classified as HER2-positive versus HER2-negative based on a combination of immunohistochemistry (IHC) and in situ hybridization (ISH) techniques [1, 3]. Anti-HER2 agents have been reserved to patients with BC expressing high levels of HER2 protein or with *ERBB2* amplification. Recently, increasing evidence indicates that a subset of patients affected by BCs expressing HER2 on the membrane at low levels detectable by standard IHC (score 1+ or 2+) but lacking *ERBB2* amplification can respond to novel antibody-drug conjugates (ADCs) targeting HER2 [4–6]. The presence of HER2 on the plasma membrane offers an anchor to ADCs, which enter the cell and exert tumor killing [1].

At present, HER2-low BC (HLBC) is mainly an operational term. Although the current IHC-based classification allows identifying a potentially clinically targetable disease, whether HLBC constitutes a specific, biologically meaningful BC class remains to be demonstrated. HLBCs account for up to 55% of all BCs and are more frequently estrogen receptor (ER)-positive [7]. These tumors preferentially belong to the luminal molecular subtypes and harbor higher *ERBB2* mRNA levels compared to HER2-negative tumors [8, 9]. We [10] and others [9] have demonstrated that the HER2-enriched subtype can be encountered in HLBCs. Of note, this seems to happen at a significantly higher frequency compared to HER2-negative BCs.

Here, we aimed at characterizing HLBCs at the molecular level to assess whether they represent a distinct entity. Specifically, we (i) charted the mutational and transcriptional landscape of a large cohort of well-characterized HLBCs, (ii) revealed a possible heterogeneity among HLBCs stemming from genomic features, and (iii) compared the genomic landscape of HLBCs with HER2-positive and HER2-negative BCs.

## Methods

### Cohorts

**Fig. 1:**
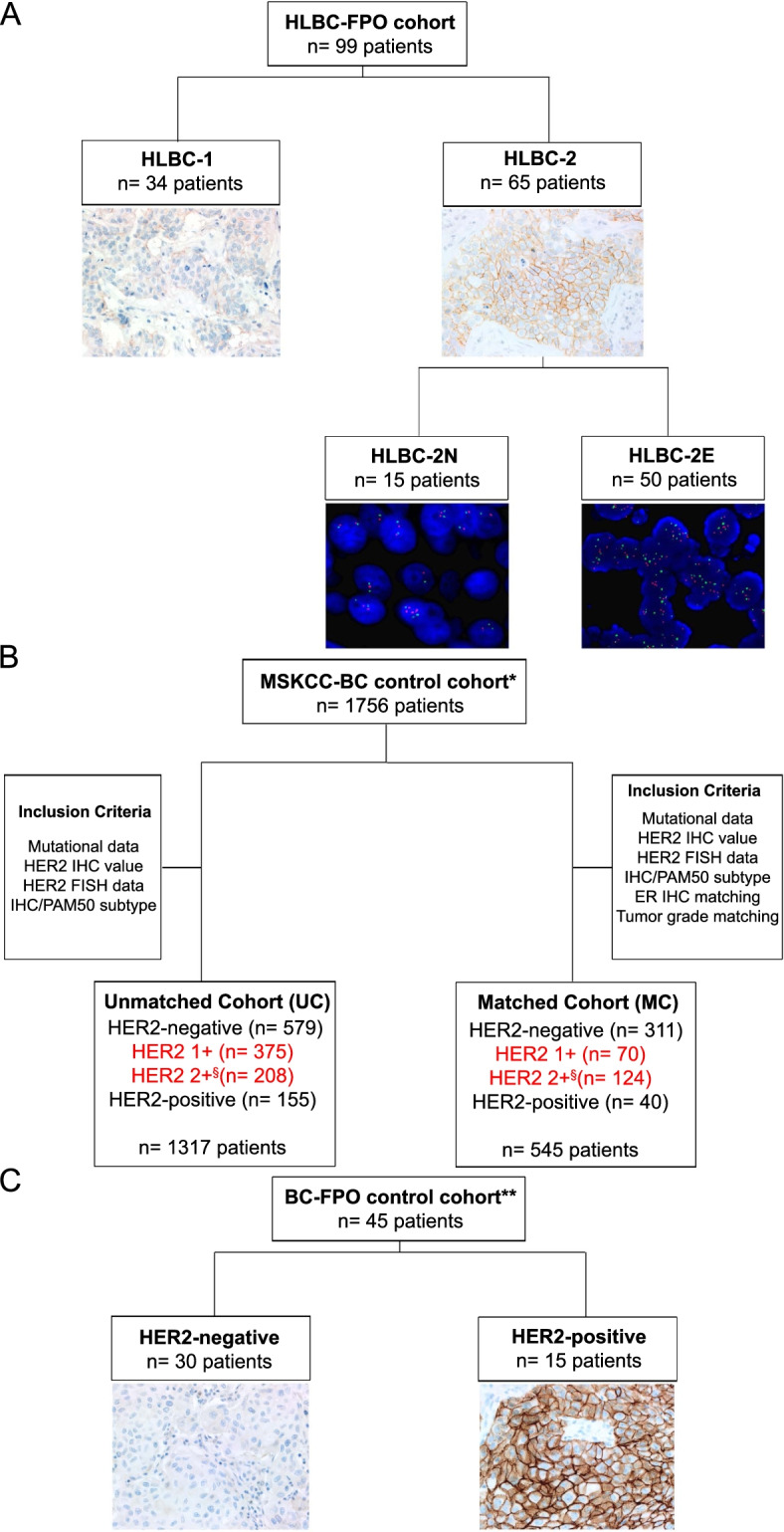
Description of the study cohorts. **A** Consort diagram of the HER2-low breast cancer (HLBC)-FPO cohort. A total of 99 patients were included, characterized by an IHC score 1+ (*n* = 34) or 2+ and FISH non-amplified (*n* = 65). HLBC-2 comprised lesions harboring a *ERBB2* copy number (CN) < 4 and a ratio *ERBB2*/CEP17 ≤ 2 (FISH-negative, HLBC-2N, *n* = 15) and tumors with *ERBB2* FISH results in the equivocal range (ratio *ERBB2*/CEP17 < 2, CN ≥ 4 and < 6, HLBC-2E, *n* = 50). **B** Consort diagram of the public Memorial Sloan-Kettering Cancer Center (MSKCC) BC cohort, which was exploited for comparison of mutational landscapes. The MSKCC BC cohort contains (i) discrete HER2 IHC scores, (ii) gene mutations identified with a DNA-based targeted panel, and (iii) histopathological data (ER status, histological grade). From 1756 samples, we derived two populations: (i) the unmatched cohort including 1317 annotated samples and (ii) the matched cohort, which contains 545/1317 samples matched by ER status and histological grade of the HLBC-FPO cohort. The detailed inclusion criteria are described in the “Methods” section and Additional file 1: Supplementary Methods section; a summary of the criteria is illustrated in the figure. **C** Consort diagram of the control cohort for RNA-based analyses. A control cohort of BC was retrieved from our archives (*n* = 45), composed of HER2-negative (score 0, *n* = 30) and HER2-positive (score 3+, *n* = 15). These cases were profiled by the NanoString BC360 panel, similarly to the HLBC-FPO cohort. Legend: *public control cohort for DNA data comparison; ^§^cases score 2+ *ERBB2* not amplified; red colored text for HLBC-MSKCC cases; **internal control cohort for RNA data comparison

We collected 99 early BC patients affected by tumors lacking *ERBB2* amplification (*ERBB2*/CEP17 ratio < 2, *ERBB2* copy numbers < 6 by FISH analysis) and showing either a score of 1+ or 2+ in IHC (herein named HLBC-FPO cohort, Fig. 1A). IHC stainings (4B5 assay by Roche-Ventana, Tucson, USA) were re-assessed based on the ASCO-CAP guidelines [3]. The final cohort was subdivided into three IHC-based categories: 34 samples HER2 score 1+ (HLBC-1), 15 samples HER2 score 2+ with *ERBB2* copy number < 4 that is equal to negative HER2 status (HLBC-2N), and 50 samples HER2 score 2+ with *ERBB2* copy number in the equivocal range between 4 and 6 (HLBC-2E) (Fig. 1A).

This is a series of non-consecutive archival formalin-fixed paraffin-embedded (FFPE) primary carcinomas. The cases were selected based on (i) having optimal tissue fixation (to avoid biases in the definition of HER2-low category) and (ii) showing adequate cellularity, i.e., more than 90% of tumor cell content after mesodissection (to allow downstream analyses).

The cases were collected in the frame of a prospective protocol approved by the “Istituto di Candiolo FPO-IRCCS” Ethical Committee (“Profiling” #001-IRCC-00IIS-10, ClinicalTrials.gov Identifier: NCT03347318 [11], see Declarations section). Details about the survival data (in terms of relapse-free survival) and type of adjuvant treatment (endocrine therapy, chemotherapy) were collected for all of the patients regularly followed up at our institution.

The study includes two sets of control cohorts. To compare the mutational landscape of HLBC-FPO with HER2-negative and HER2-positive BCs, we exploited publicly available targeted sequencing data from the Memorial Sloan-Kettering Cancer Center (MSKCC cohort [12], Fig. 1B). We actually screened all the BC open datasets of gene mutations available on cBioportal (last access date 2021 December 31) [13]. To allow a balanced comparison, the control set needed to contain (i) score-based evaluation of HER2 IHC data for the HER2-low group and subgrouping definition, (ii) mutational data generated from a DNA-based targeted panel, and (iii) clinico-pathological data to match the features of the HLBC-FPO cohort (at least information about ER status and histological grade). By taking into account the previous parameters, the MSKCC cohort resulted the best-suited publicly available control cohort.

Conversely, since for gene expression analysis we used a NanoString approach that is not been widely adopted, we profiled a series of HER2-negative (*n* = 30) and HER2-positive (*n* = 15) BCs selected from our archives and collected in the frame of the “Profiling” protocol quoted above for HLBCs (Fig. 1C and Additional file 1: Supplementary Methods).

### DNA-based targeted sequencing

DNA extracted from the 99 formalin-fixed paraffin-embedded (FFPE) samples (80 ng) was subjected to deep sequencing using the TruSight Oncology (TSO) 500 panel (Illumina, San Diego, USA; 523 genes, size: 1.94 Mb), following the manufacturer’s protocol, which assesses microsatellite instability (MSI) status (120 loci), tumor mutation burden (TMB), and copy number (CN) data (59 genes). The choice of a targeted panel was based on the robustness of the performance of this technique on DNA extracted from FFPE samples.

Libraries were sequenced on a NovaSeq 6000 instrument (Illumina) to reach a minimum of 500× read depth. Data processing is described in Additional file 1: Supplementary Methods. Actionability of the mutations was annotated with OncoKB [14] and the ESMO Scale for Clinical Actionability of Molecular Targets (ESCAT) [15, 16]. Mutational signatures were assessed as previously reported [17]. The TMB was assessed by the local app as the ratio between the total somatic, non-synonymous variants with a variant allele frequency (VAF) > 0.05 and the sequenced genome for each sample, as reported here [17]. InterVar pipeline [18] and ClinVar significance [19] were used to assess the germline pathogenic variants in BC susceptibility genes [20]. The level of pathogenicity was annotated by using the ANNOVAR tool [21].

### Assembly of the control cohort for comparison of mutational landscapes

Mutational data were compared to the public MSKCC BC cohort [12], which contains (i) discrete HER2 IHC scores, (ii) gene mutations identified with a DNA-based targeted panel with wet and bioinformatic conditions similar to the one employed in our study, and (iii) histopathological data (ER status, histological grade) (see also Additional file 1: Supplementary Methods).

Molecular and histopathological data were downloaded from cBioPortal [13]. From 1756 samples, we derived two populations: (i) the unmatched cohort including 1317 annotated samples and (ii) the matched cohort, which contains 545/1317 samples matched by ER status and histological grade of the HLBC-FPO cohort. Hence, each group of the control cohort was composed of 90% of ER-positive and 10% of ER-negative samples; in addition, the ER-positive samples comprised 55% of high-grade tumors (G3), and the ER-negative samples were composed of 80% of G3 tumors (Fig. 1B).

### Targeted gene expression profiling

RNA-targeted gene expression profiling (tGEP) was carried out on 91 HLBCs, 30 HER2-negative, and 15 HER2-positive samples using the BreastCancer 360™ Panel (NanoString Technologies, Seattle, USA, 776 BC-related genes), following the manufacturer’s protocol. The nSolver Software (NanoString Technologies) with the nSolver Advanced Analyses calculated the pathway score as the first principal component of the pathway genes normalized expression, the cell type score [22], and the differential gene expression (DGE) analysis, performed with respect to covariates (ER status, IHC subtype, and tumor grade, “G”) (Additional file 1: Supplementary Methods).

On the tGEP data, we also applied the consensus-based non-negative matrix factorization (NMF) algorithm to devise a class discovery experiment by using the R tool “NMF” [23]. We selected the most variable genes (top 33% genes), and NMF was carried out by using a predetermined number of clusters (K), which varied from 2 to 6. Since this experiment was run to identify a novel classification system based on the different clusters stemming from the NMF analysis, we selected the clustering size by (i) ranking the best cophenetic coefficients and (ii) discarding clustering overlapping the PAM50 intrinsic subtyping [24], to identify new RNA-based classes.

After class discovery, nSolver Advanced Analysis was re-applied to evaluate the differential gene expression analysis between the classes.

### Orthogonal validation

MSI data were confirmed by IHC of the mismatch repair (MMR) proteins and by using a PCR-based assay. Selected CN alterations (CNA, affecting *KRAS*, *MET*, *FGFR1*, *CCND1*, *ERBB2*) were investigated by FISH. Details are available in the Additional file 1: Supplementary Methods.

### Statistical methods

Statistics were assessed by using the R software v4.03. Distribution differences were assessed by using an unpaired *T*-test and contingency by Fisher’s exact test, always with FDR adjustment. *P*-values were considered significant when *P* < 0.05.

## Results

### HLBC-FPO cohort

The histopathological features of the HLBC-FPO series are summarized in Additional file 2: Table S1. The large majority were ER-positive BCs (88/99, 88.9%; range: 65–99%), and no ER-low [3] carcinomas were observed. Half of the series (54.5%) was composed of G3 carcinomas, and 68% of cases showed Ki67 values above 20%.

The IHC surrogate molecular subtype included luminal-B tumors (81/99, 81.8%) followed by luminal-A and triple-negative carcinomas (both 9/99, 9.1%). PAM50 subtyping was available for 89/99 cases, of which 29 were luminal-A (29.3%), 50 luminal-B (50.5%), four HER2-enriched (4.1%), and six basal-like tumors (6.1%).

### The genetic make-up of HLBCs

**Fig. 2:**
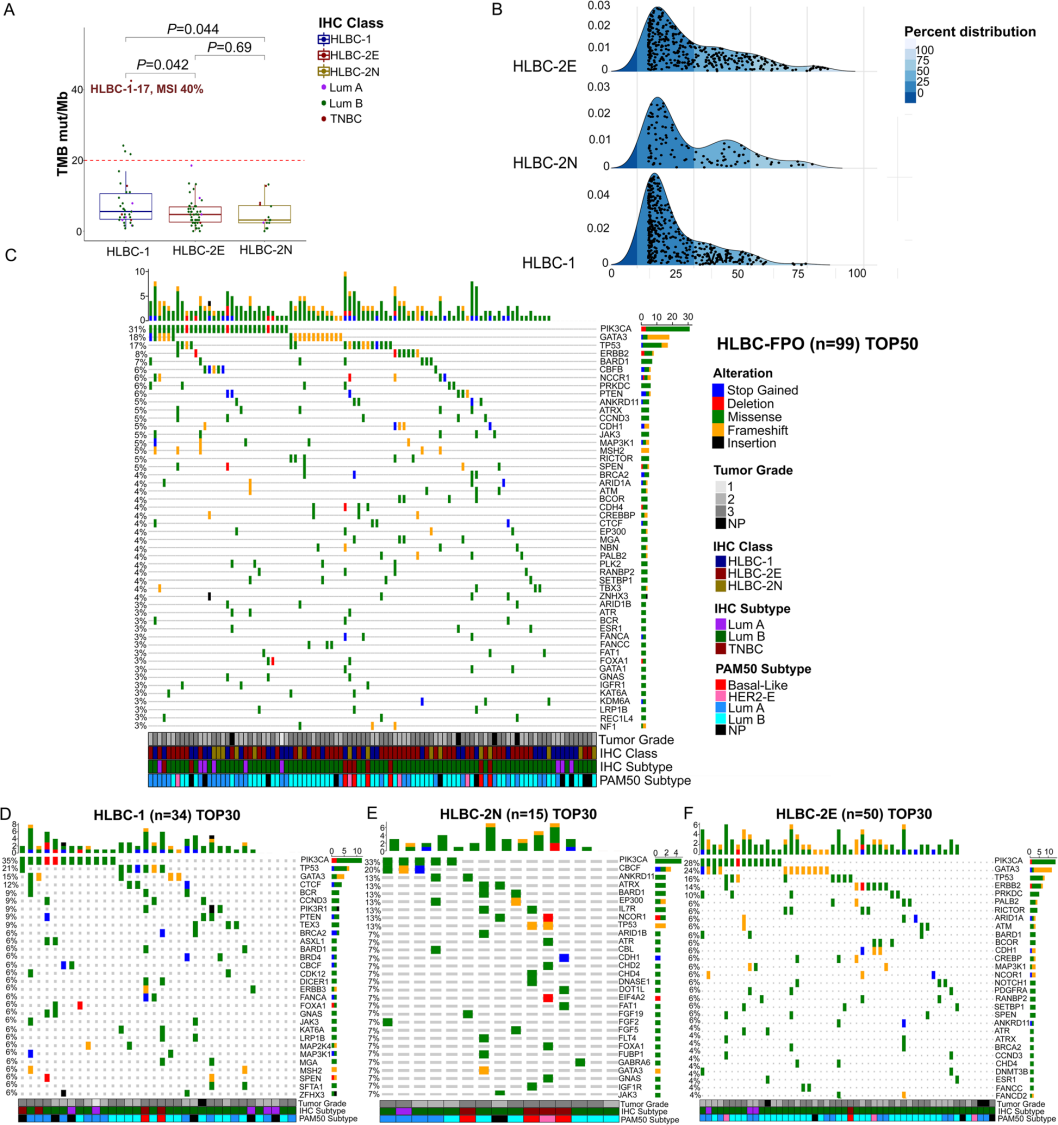
Mutational landscape of HLBC-FPO cohort. **A** Boxplots of TMB values for each HLBC subgroup. Dots are color-coded according to the intrinsic molecular subtype. One case (HLBC-1-17) harbored high TMB (42.52) and MSI (40%). Out of 34 HLBC-1 cases, four showed TMB values above 20 and eight between 10 and 20 mut/Mb. Four cases (4%) were wild-type for all the analyzed genes. **B** VAF distributions across the HER2 subgroups. Each dot represents a mutation. Blue sections divide the plots into 25-percentile VAF ranges. **C** OncoPrint of the 99 HLBC-FPO tumors subjected to targeted sequencing, reporting the 50 most frequently mutated genes. Gene names and relative frequency of mutations are reported in the double *y*-axis. Top: the bar graph defining the number of variant/patient for the selected genes; bottom: annotation for tumor grade, IHC class, IHC subtypes, and PAM50 subtypes. **D**–**F** OncoPrint of the HLBCs-1, 2E, and 2N showing variants in the 30 most frequently mutated genes. TMB, tumor mutation burden; VAF, variant allele frequency. The legend for all the OncoPrints and annotations is reported in **C**

The mean read-depth for the 99 sequenced samples was 523× (Additional file 3: Table S2). The average TMB value was 6.24 mutations per megabase (mut/Mb) (range: 0–42.52). HLBC-1 carcinomas showed significantly higher TMB values (8.46, 95%CI = 5.47–11.46) with a sub-clonal variant allele frequency (VAF = 0.175, 95%CI = 0.15–0.18) compared to HLBCs-2N (TMB = 4.70, 95%CI = 2.4–6.99, *P* = 0.04; VAF = 0.244, 95%CI = 0.21–0.28, *P* < 0.01, unpaired *T*-test) and HLBCs-2E (TMB = 5.18, 95%CI = 4.12–6.24, *P* = 0.04; VAF = 0.256, 95%CI = 0.23–0.27, *P* < 0.01 unpaired *T*-test) (Fig. 2A, B). When evaluating the most superimposable COSMIC mutational signatures [25, 26], the HLBC cohort showed four main signatures (signatures 1, 5, 13, and 20). HLBC-2N and HLBC-2E cohorts showed the same mutational features (signatures 1, 5, and 13), whereas the HLBCs-1 showed one additional private signature (signature 30) (Additional file 4: Fig. S1).

The most frequently mutated genes were *PIK3CA* (31/89, 33%), *GATA3* (18/99, 18%), *TP53* (17/99, 17%), and *ERBB2* (8/99, 8%) (Fig. 2C). We also identified 10 somatic mutations in hereditary BC-associated genes (*BARD1*, *BRCA2*, *PALB2*). From a germline standpoint, we detected two pathogenic variants (one affecting *BRCA*1 and one affecting *BRCA2*, 2/99, 2%), which were clinically confirmed; in addition, 14 variants with uncertain significance (VUS) in 10 BC susceptibility genes were observed, the most interesting being a *TP53* missense mutation, reported as likely pathogenic for a breast neoplasm-associated syndrome (Additional file 5: Table S3).

When stratifying the results by HLBC subgroups, *PIK3CA* remained the most frequently mutated gene across all the groups, whereas *TP53* was the second most mutated gene only in HLBCs-1 (21% vs 17% overall) (Fig. 2D). *ERBB2* mutations were restricted to the HLBC-2E group (Fig. 2E), and the HLBC-2N group was the most variable: lower frequency of *TP53* and *GATA3* mutations and *CBFB* as the second most prevalent mutated gene (3/15, 20%) (Fig. 2F).

**Fig. 3:**
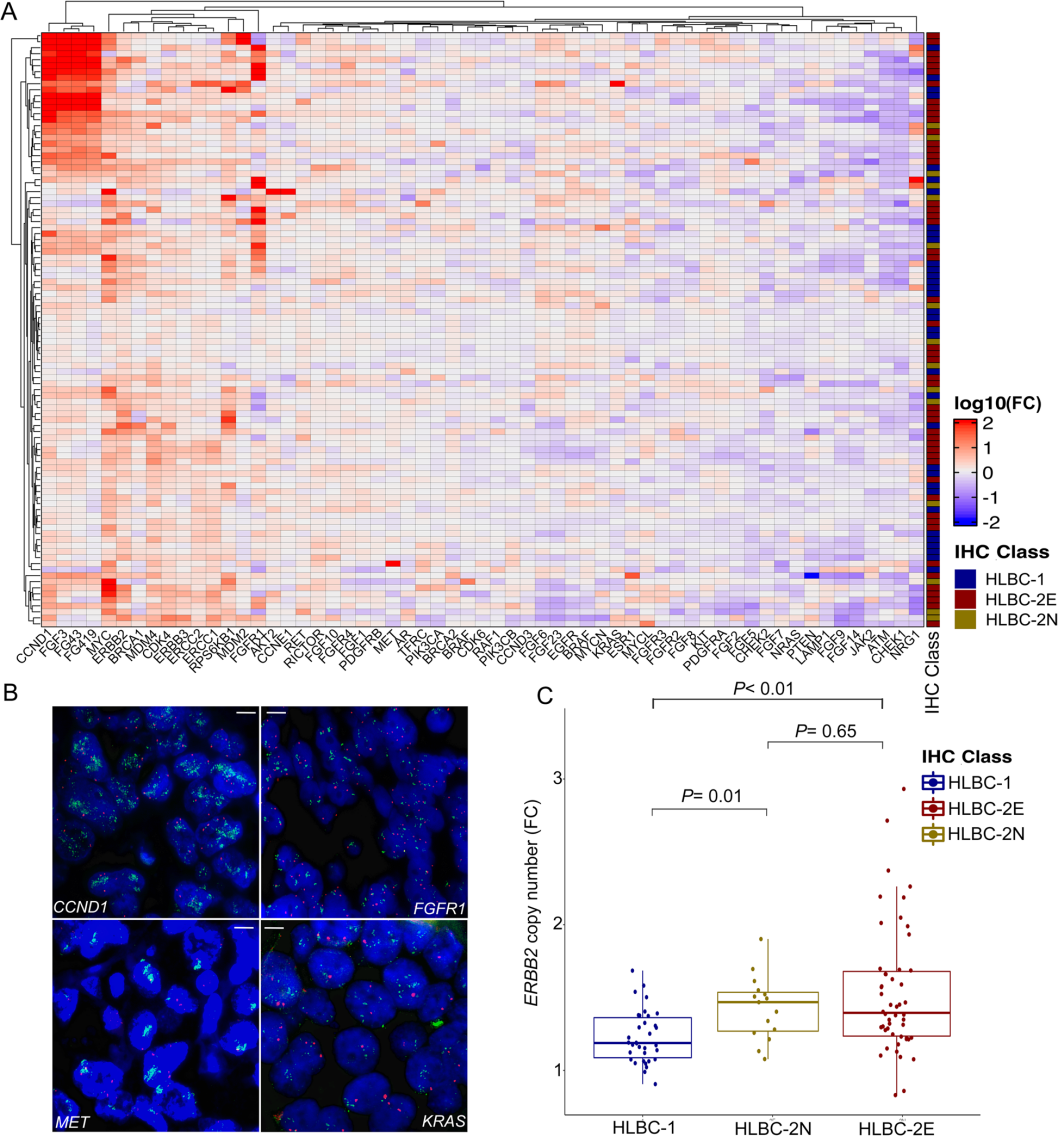
Copy number alterations across the HLBC-FPO cohort. **A** Heatmap of the copy number alteration (CNA) for the 59 genes comprised in the panel, reporting the log10 value of the fold change (FC), with a blue-to-red map describing gene loss-to-gain. Raw dendrogram groups patients by similar CNA, and column dendrogram groups co-occurrent CNAs. IHC class annotation on the right-hand side. **B** Validation of CNA results by FISH. Clockwise, *CCND1*, *FGFR1*, *MET*, and *KRAS* gene amplification (green signals: gene; red signals: centromeric probes). White bar: 5-μm in size. **C** Boxplot of the *ERBB2* gene FC distribution (sequencing data) for the IHC-based cohorts. HLBCs-2 showed a significantly higher number of *ERBB2* copies compared to HLBCs-1, with the highest values identified in the HLBC-2E cohort, in line with the FISH equivocal subtyping (HLBC-1 vs HLBC-2N *P* = 0.01, HLBC-1 vs HLBC-2E *P* < 0.01, unpaired *T*-test)

We then turned to CN changes. We detected 12 genes with CN-gain in 25/99 patients (25%) and 5 genes with CN-loss in 10/99 patients (10%). The most frequently CN-gain affected *CCND1* (11%, 11/99), *FGF3*, *FGF19*, *FGF4*, and *FGFR1* (8/99 samples, 8%, Fig. 3A). One sample showed a *PTEN* homozygous deletion, whereas 5/99 (5%) patients showed an *NRG1* hemizygous deletion. DNA FISH analysis orthogonally validated *CCND1*, *FGFR1*, *MET*, and *KRAS* amplifications (Fig. 3B). For each gene, we plotted the CN fold change values in the distinct HLBC subgroups. None of the genes with CN changes was differentially altered among the groups. Of note, the HLBCs-2 harbored a significantly higher *ERBB2* CN compared to the HLBC-1 group, with the highest level of *ERBB2* CN identified in the HLBCs-2E (Fig. 3C).

### Mutational landscape: HLBCs versus HER2-positive and HER2-negative carcinomas

**Fig. 4:**
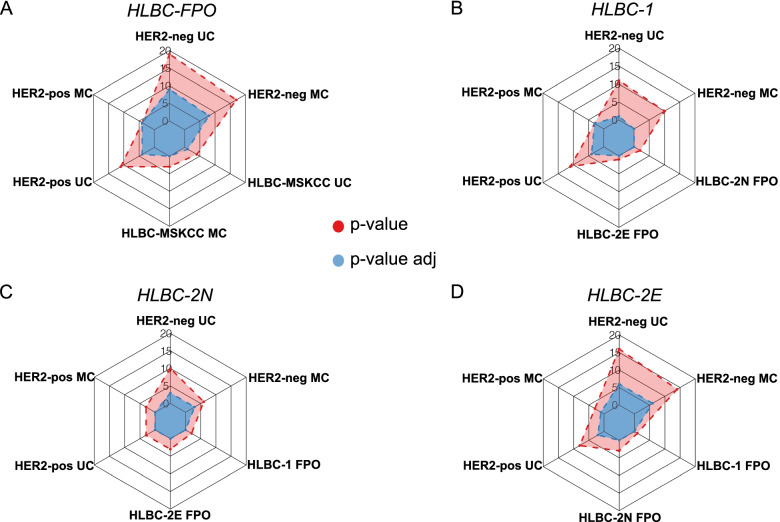
Comparison of the mutational landscape between HLBC-FPO and MSKCC control cohorts. Spider plots depicting multiple comparisons. On the *y*-axis of the spider plot, we report the number of genes with a significantly different number of mutations (multiple Fisher’s exact test with FDR adjustment) between the reference group and each control group, for both *P*-value (red) and adjusted *P*-value (blue). **A** Spider plot comparing the HLBC-FPO group and the MSKCC BC (unmatched and matched) control cohorts. For instance, when considering the whole HLBC-FPO cohort, we detected 19 differentially mutated genes with respect to the HER2-negative unmatched cohort and 16 for the HER2-negative matched cohort. The same comparisons run after FDR adjustment resulted in 9 and 8 differentially mutated genes. When the whole HLBC-FPO cohort was compared with HER2-positive tumors, we detected 11 differentially mutated genes with respect to the unmatched cohort and 4 for the matched cohort. The same comparisons run after FDR adjustment resulted in 4 differentially mutated genes for both. **B** Spider plot comparing the HLBC-1 group, the HLBCs-2E and 2N, and the MSKCC BC (unmatched and matched) control cohorts. **C** Spider plot comparing the HLBC-2N group, the HLBCs-1 and 2E, and the MSKCC BC (unmatched and matched) control cohorts. **D** Spider plot comparing the HLBC-2E group, the HLBCs-1 and 2N, and the MSKCC BC (unmatched and matched) control cohorts

Next, we wondered if HLBCs show a differential pattern of mutated genes compared to HER2-negative and HER2-positive tumors. First, we assessed the quantitative differences among the cohort. We elaborated a control HLBC-MSKCC cohort superimposable with the HLBC-FPO cohort for ER status, G, and for relative composition of score 1+ and score 2+ cases (see the “Methods” section). The HLBC-FPO and the HLBC-MSKCC cohorts were superimposable in terms of mutated genes. Next, we elaborated control cohorts of HER2-negative and HER2-positive that were either matched or unmatched for major histopathological features. Considering the whole HLBC-FPO cohort, we detected multiple differentially mutated genes compared to both HER2-negative and HER2-positive BCs (Fig. 4A). To deconvolute the heterogeneity within HLBCs, we performed the same analysis by stratifying for IHC subgroups. HLBCs-1 were more like HER2-negative BCs than HLBCs-2N and HLBCs-2E (0 vs 3 vs 6 differentially mutated genes compared to the HER2-negative matched cohort, Fisher’s exact test FDR adjustment). Conversely, compared to HER2-positive carcinomas, HLBC-FPO score 2+ cases showed a very limited number of differentially mutated genes (2N = 0 and 2E = 1, Fisher’s exact test FDR adjustment), whereas HLBC-1 showed 4 differentially mutated genes (Fig. 4B–D, Additional file 6: Table S4). By examining the differentially mutated genes, three were of particular interest in breast cancer: (i) the *ESR-1* gene was significantly more frequently mutated in HER2-negative tumors, (ii) *TP53* mutations were significantly more frequent in HER2-positive tumors, and (iii) the *SPEN* gene was more frequently mutated in HLBCs, in comparison with both HER2-negative and HER2-positive carcinomas.

As a further analysis, we also compared the variant distribution among the cohorts in terms of both variant type (VT, e.g., the type of mutation at the DNA level) and variant classification (VC, e.g., the type of mutation at the protein level). Next, we annotated the pathogenicity level (grouped in 4 classes: nonsense, damaging, potentially damaging, and tolerated) of the variants in the genes identified as differentially mutated in the comparisons between the HLBC-FPO and the matched control cohorts (those genes reported in the spider plots of Fig. 4).

In terms of the type of variants, Bonferroni correction of the chi-square test revealed the differences in terms of both VC and VT only between HER2-negative (both MC and UC) and HLBCs (*P*-values reported in Additional file 7: Table S5), mainly due to a reduced relative contribution of SNVs and missense variants in the HER2-negative control cohort (Additional file 4: Fig. S2A-B). When annotating for pathogenicity, no differences were identified comparing HLBCs and HER2-positive carcinomas, whereas HLBCs showed significant differences with HER2-negative MCs, with more damaging mutations of the differentially mutated genes affecting HER2-negative disease (*P* < 0.01, Bonferroni correction of the chi-square test; Additional file 4: Fig. S2C; Additional file 8: Table S6). Of note, the latter category was also enriched for *ESR-1* mutations compared to HLBCs, as reported above.

Potential differences across cohorts were also assessed for copy number alterations: the HLBC cohorts overlapped also in terms of gene CN gains, whereas *FGFR1* gains were specific to the HER2-negative cohort (15% vs 8% of HLBCs, *P* = 0.04, Fisher’s exact test). As expected, the *ERBB2* gene was significantly more amplified in the HER2-positive cohort (100% vs 0% in HLBCs, *P* < 0.01, Fisher’s exact test). All *P-*values are reported in Additional file 9: Table S7.

### The landscape of actionable alterations in HLBCs

**Fig. 5:**
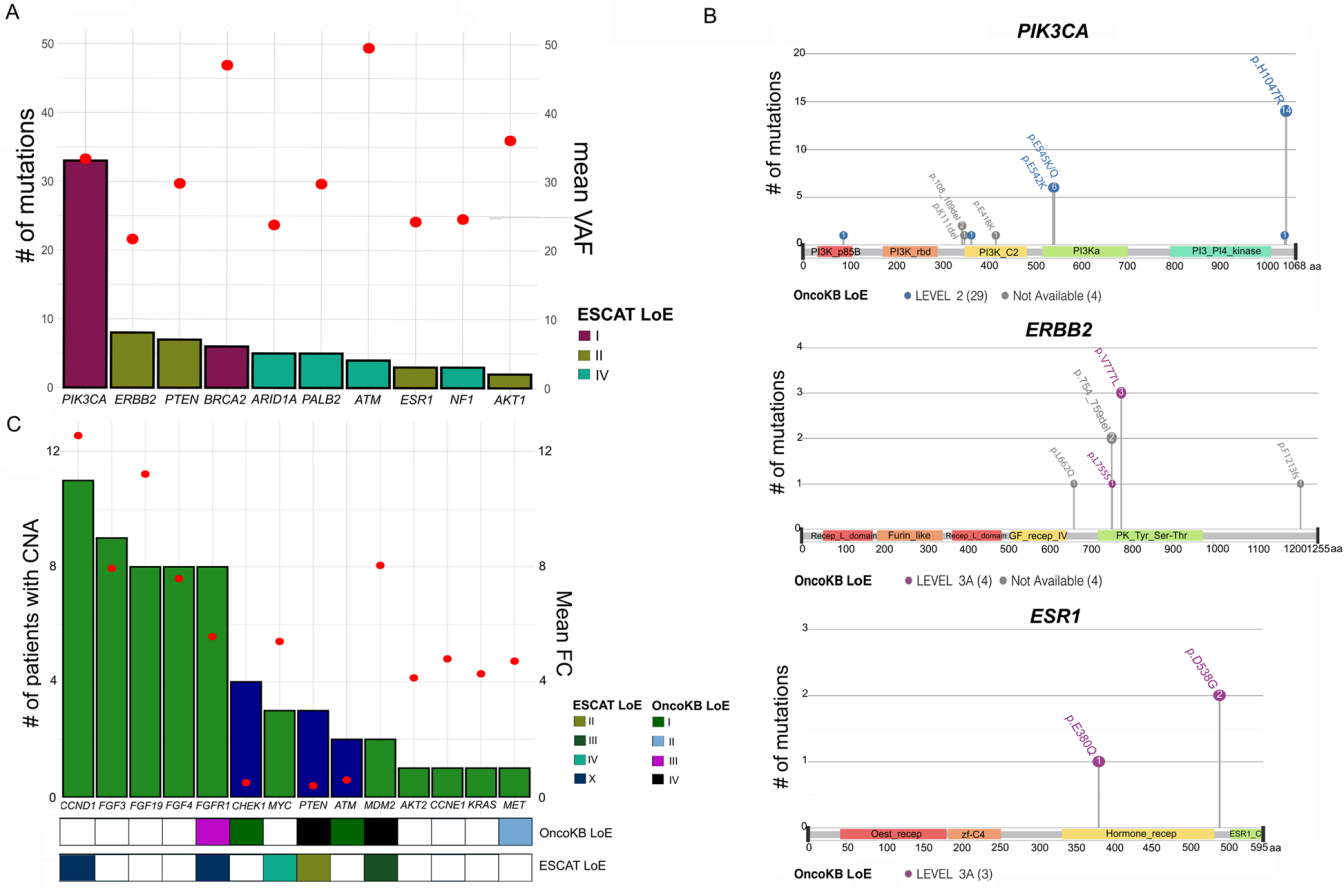
Actionability of the mutations in HLBCs. **A** Combined, double *y*-axis dot/barplot reporting the identified variants with OncoKB/ESCAT level of evidence (LoE). The left *y*-axis (barplot) reports the number of mutations per gene. The right *y*-axis shows the mean VAF level of these mutations (red dots). The bars were colored by the ESCAT LoE. **B** Lollipop plots for *PIK3CA*, *ERBB2*, and *ESR1* genes, reporting the amino acid change, the mutation type, and the OncoKB LoE. Four variants of both *PIK3CA* (3 deletions, 1 SNV of the C2-domain) and *ERBB2* (2 deletions, 1 SNV, and 1 non-sense mutation) showed no LoE. Gray dots define the mutations with unknown actionable significance. **C** Combined, double *y*-axis dot/barplot reporting the CNA genes with OncoKB oncogenic behavior in this cohort. The left *y*-axis (barplot) reports the number of patients with CNA per gene. Green bars depict the genes with CN gain, and blue bars depict those with CN loss. The right axis shows the mean fold change (FC) of these copy number alterations (CAN, red dots). Annotation at the bottom defines the available OncoKB/ESCAT LoE

Forty-three single nucleotide variants (SNVs) and seventeen CN alterations were classified as druggable [14–16]. We identified 76 SNVs with available OncoKB/ESCAT level of evidence (LoE) from 10 genes. For 45 variants, OncoKB assigned LoE in 40 patients (40%). *PIK3CA* mutations were the most pervasive alteration, with 33 annotated variants with a mean VAF of 0.33 (Fig. 5A); of these, 28/33 had a level of evidence of 2 (Fig. 5B). The second most annotated gene was *ERBB2*, with 4/8 mutations reported to be non-actionable (Fig. 5B). We also detected two *ESR1* mutations, both with a 3A LoE (Fig. 5B) and three somatic variants in familial BC genes, reported to respond to PARP inhibitors (two *BRCA2* and one *PALB1* variants) (Additional file 10: Table S8).

When considering OncoKB/ESCAT annotated CN alterations, we detected 53 CN gains and 9 CN losses in 14 genes across 62 samples. OncoKB reported a LoE for five genes, with *FGFR1* being the most prevalent (8 patients) and a *MET* CN gain with a LoE 2 (Fig. 5C). ESCAT annotation reported a LoE IV for *MYC* amplification, absent in the OncoKB annotation.

One case showed a very high TMB and MSI. Retrospective evaluation of the clinicopathological features of this sample revealed a triple-negative phenotype, an intense immune infiltrate (tumor-infiltrating lymphocytes, TILs = 90%), and a lack of MLH1-PMS2 expression (Additional file 4: Fig. S3).

Overall, by considering at least one alteration with an OncoKB/ESCAT LoE, 52 patients (52.5%) showed a potentially actionable alteration.

### Transcriptomic analysis reveals a spectrum of diversity within HLBCs

Next, we explored whether transcriptomics can further expand the molecular classification of HLBCs, by comparing the gene expression profiles (GEPs) of 91 HLBCs with control cohorts of 30 HER2-negative and 15 HER2-positive carcinomas.

We defined different and similar traits between HLBCs and controls by calculating both a global significance score (GSS) and a direct GSS (dGSS, Additional file 1: Supplementary Methods) within the following approach: (i) independent differential gene expression (DGE) with HER2-negative or HER2-addicted tumors as the reference group, (ii) GSS heatmap of HLBCs and the non-reference group, (iii) identification of the most variable gene sets shared or private for HLBCs and the non-reference group. These analyses allowed to evaluate the similarity between HLBCs and HER2-negative BCs by assessing how dissimilar they were from HER2-positive BCs and vice versa.

**Fig. 6:**
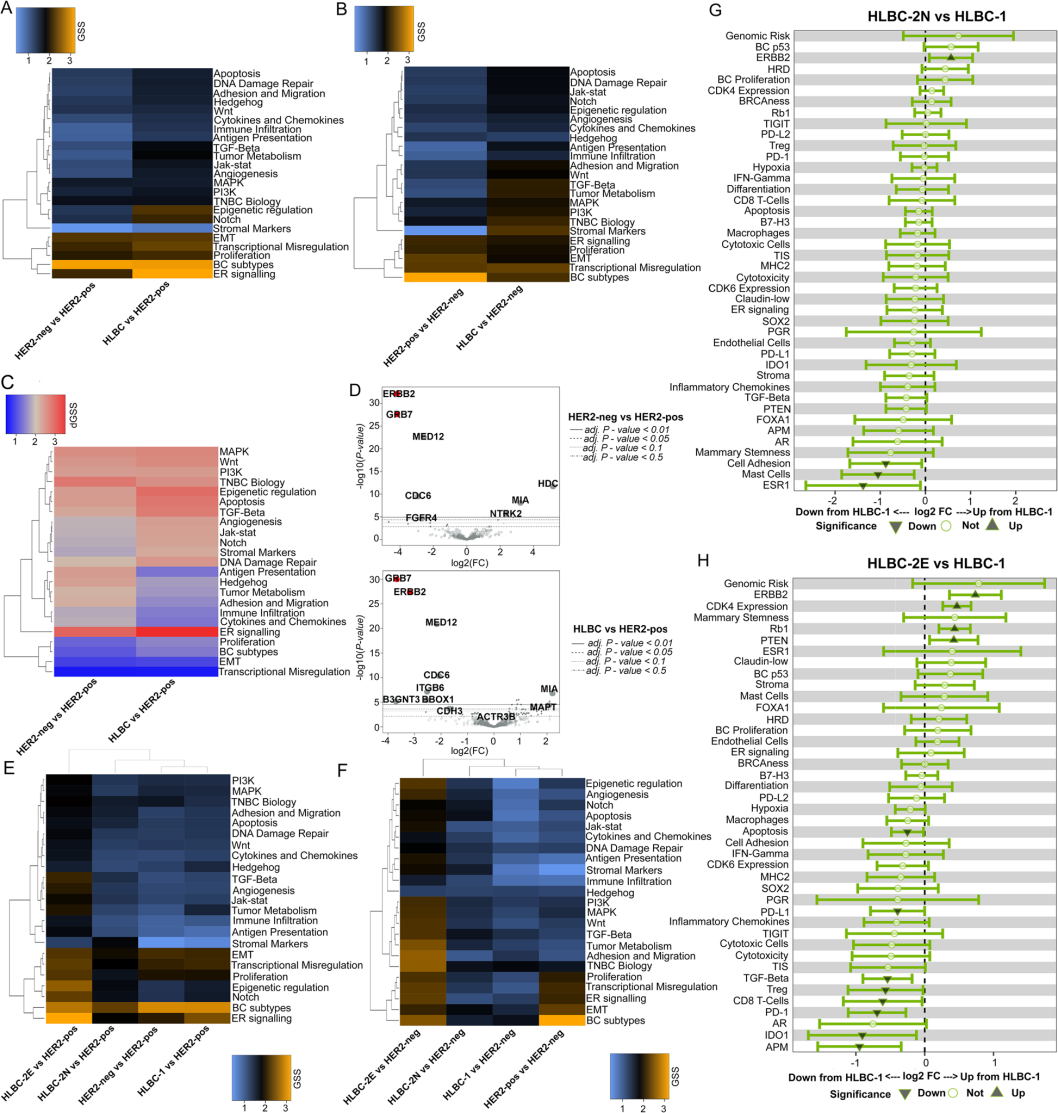
Differential gene expression (DGE) analysis of HLBC subsets. **A**, **B** Inter-group DGE analysis with the HER2-positive (**A**) and with HER2-negative (**B**) carcinomas as a reference. The heatmap reports the GSS as a variable: blue pathways with no differential expression and orange those with high differential expression. **C** Heatmap of the dGSS, reporting the polarization (up or down) of differential expression (HER2-positive as the reference group). **D** Volcano plots reporting the DE genes between HER2-negative and HLBCs versus HER2-positive tumors. On the *y*-axis, we reported the -log10 of the *P*-value (ANOVA model, FDR-adjusted), and on the *x*-axis, the log2 fold change. Red squares represent the *ERBB2* and *GRB7* gene expression. **E**, **F** Inter-group DGE analysis after stratification for HER2 subgroups (HER2-positive carcinomas as a reference in **E** and HER2-negative as a reference in **F**). The heatmap reports the GSS as a variable: blue pathways with no differential expression and orange those with high differential expression. **G**, **H** Inter-HLBC DGE analysis, with HLBC-1 class as reference and HLBC-2N (**G**) or HLBC-2E (**F**) as a target. The *y*-axis reports the gene sets (NanoString private algorithm), and *x*-axis reports the differential expression means (95%CI) between response variables (unadjusted scale). The dashed vertical axis is shown at fold change equal to zero, indicating equivalent expression between the groups. A signature is considered significant if the 95%CI (the horizontal line) does not cross the vertical axis representing the baseline group and therefore no difference to that baseline group. Dark green triangles marked the differentially expressed gene sets

Taking HER2-positive tumors as a reference, HLBCs and HER2-negative BCs presented similar differentially expressed gene sets (Fig. 6A). HLBCs displayed private gene sets compared to HER2-positive carcinomas, but with lower GSSs. The dGSS heatmap highlighted the upregulation of the ER signaling, which represented the main differentiation feature from HER2-positive versus both HLBCs and HER2-negative tumors (Fig. 6C).

Conversely, when HER2-negative BCs were used as a reference, HLBCs showed several gene sets with an intermediate GSSs, only partially overlapping with HER2-positive BCs (Fig. 6B). This suggests that HLBCs are different from HER2-negative tumors for similar gene sets yet more heterogeneous compared to the gene sets characterizing HER2-positive BCs. In the latter, *ERBB2*- and *ERBB2*-coamplified genes [27, 28] were the main driver of differential gene expression. HLBCs showed increased expression of *ERBB2* but not of *ERBB2*-related genes (Fig. 6D).

By applying the same type of analysis to the distinct HLBC subgroups, we observed that HLBCs-1 showed overlapping differentially expressed gene sets with HER2-negative tumors, whereas HLBC-2E was the most independent group and HLBC-2N represented an intermediate category with intermediate GSSs. These features were observed in both comparisons with HER2-negative and HER2-positive BCs (Fig. 6E, F).

Lastly, we performed the same analysis using HLBC-1 as a reference. HLBC-2E cases showed an increased *ERBB2* expression, which was only partially retained in HLBC-2N tumors (Fig. 6G, H). HLBC-2E also showed upregulation of *CDK4*, *PTEN*, and *RB1* genes. Conversely, HLBC-1 showed increased signatures associated with immune infiltrate, such as expression of *PD1*, *PD-L1*, *IDO-1*, and *APM*, but only when compared with the HLBC-2E (Fig. 6G, H). These results confirm a strong independence of HLBC-2E tumors from all the other groups, with more heterogeneity for the HLBC-1 and HLBC-2N groups.

### Four transcriptionally distinct HLBC subgroups: the LAURA classification

Unsupervised, hierarchical clustering of pathway scores demonstrated a lack of IHC-driven clusters (Additional file 4: Fig. S4A), whereas we observed enrichment of specific signatures in samples regardless of the HLBC class (Additional file 4: Fig. S4B-C-D).

**Fig. 7:**
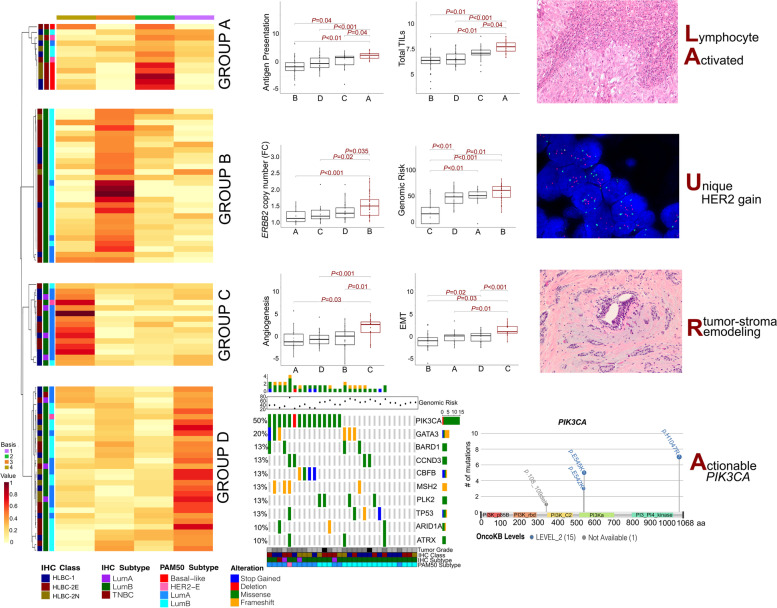
NMF cluster discovery for the LAURA classification. The non-negative matrix factorization (NMF) approach identifies four metagenes (signatures) summarized in the expression profile matrix. Each column corresponds to a sample, scaled to the sum of one of the signatures identified by the NMF, and ordered by clusters. The 4 clusters are annotated with respect to IHC class, IHC, and PAM50 subtypes and by the color map of their associated metagenes. For each cluster, specific features are reported, in the form of boxplots, OncoPrint, lollipop plot, H&Es, and FISH images. The ontological derived terms describing the main features of each group are reported on the right-hand side leading to the LAURA acronym for the classification: LA, lymphocyte activated; U, unique HER2-gain; R tumor-stroma remodeling; A, actionable *PIK3CA*

We investigated whether the gene expression could unveil alternative and significant subgroups for HLBCs. To this end, we applied the NMF algorithm to cluster 85 out of 91 samples for which all the clinico-pathological and molecular data were available, to find gene expression (GE)-based groups independent from the molecular subtypes. We identified four GE-based clusters (Fig. 7, Additional file 4: Fig. S5C) selecting the best NFM model (cophenetic coefficient rank in Additional file 4: Fig. S5A), based on the 33% of the most variable genes (Additional file 4: Fig. S5B). Only basal-like tumors clustered into a single group (group A) (Additional file 4: Fig. S6A), composed also of non-basal tumors (2 luminal-A, 3 luminal-B, 1 HER2-enriched). Luminal-A carcinomas were scattered across all groups (A = 3%, B = 21%, C = 35%, D = 41%), whereas luminal-B were mostly distributed into groups B (48%) and D (37%). The four HER2-enriched carcinomas were identified in all groups except group A.

To ontologically define these groups, we exploited genetic-, transcriptomic- and IHC/FISH-associated features. Group A (*n* = 12) was composed of basal- (50%, exclusive for this group) and non-basal-like (50%) tumors with a prevalence of G3 cases (11/12, 92%) and the highest Ki-67 values (mean: 64%, range: 31–96%) (Additional file 4: Fig. S6B-C). These tumors were HLBC-2N (*n* = 5) and HLBC-1 (*n* = 5), with only two HLBC-2E. Group A showed significantly higher TMB levels (Additional file 4: Fig. S6D) and exhibited an enrichment of immune infiltration phenotype compared to the other groups. The latter feature was paralleled by high TIL levels observed on H&E slides (mean value = 60.5%, range = 15–100%). All of the cases showed PD-L1 expression (> 1% by IC and > 10 by CPS).

Half of the tumors harbored *TP53* mutations (6/12, 50%), whereas a single *PIK3CA* mutation was detected (8%, Additional file 4: Fig. S6E). We refer to this group as “Lymphocyte Activated” (LA).

Group B (*n* = 28) was exclusively composed of luminal carcinomas (22 luminal-B, 6 luminal-A) homogeneously expressing ER at high levels (Additional file 4: Fig. S6F). These tumors were mainly G3 (62%), showing the highest value of genomic risk scores [24, 29] (mean: 57.61, range: 14–85) (Additional file 4: Fig. S6B, Fig. 6). Group B also displayed an enrichment of HLBC-2E tumors (78% of the total) and significantly higher values of *ERBB2* CN fold change compared to the other groups (Fig. 7). Notably, three out of the eight cases (38%) harboring a *ERBB2* mutation were included in this group. The most mutated gene in these tumors was *GATA3*, whereas a single *TP53* mutation was observed (Additional file 4: Fig. S6E). We refer to this group as “Unique HER2-gain” (U).

Group C (*n* = 15) was enriched of luminal-A tumors (11/15, 73%), characterized by the lowest Ki67 levels (mean: 21%, range: 6–64%), a high prevalence of G2 carcinomas (10/15, 67%), and the lowest Genomic Risk scores (mean: 23, range: 0–66) (Additional file 4: Fig. S6A-B, Fig. 6). Tumors of group C showed upregulation of pathways related to angiogenesis and epithelial to mesenchymal transition (Fig. 7). Moreover, gene sets of stromal markers and adhesion and migration were significantly upregulated (Additional file 4: Fig. S7A-B). Interestingly, a review of H&E slides highlighted (i) an enrichment of lobular cancers of classic type (6/12, 50%) and showed *CDH1* mutations (3/15, 20%) (Additional file 4: Fig. S6E) and (ii) a marked fibro-elastotic intratumoral stroma in the remaining cases (Fig. 7, Additional file 4: Fig. S7C). We refer to this group as “tumor-stroma Remodeling” (R).

Group D (*n* = 30) was heterogenous. This group exhibited the highest value of progesterone receptor (PgR) expression (Additional file 4: Fig. S6F); a balanced distribution of G2/G3 carcinomas (15/15), PAM50 subtypes (12 luminal-A, 17 luminal-B, 1 HER2-enriched); and HER2 groups (HLBC- = 12, HLBC-2N = 7, and HLBC-2E = 11) (Additional file 4: Fig. S6A-B, Fig. 6). These tumors showed intermediate Ki-67 expression values (mean: 30%; range: 2–78%) and genomic risk scores (mean: 49, range: 22–80), with no specific GE-based activated pathways (Additional file 4: Fig. S6C, Fig. 6). A higher prevalence of *PIK3CA* mutations was observed (15/30, 50%) compared to the overall prevalence of the 85 samples in the class discovery experiment (26/85, 31%, *P* < 0.01, Fisher’s exact test, Fig. 7, Additional file 4: Fig. S6E). Notably, 14/15 variants were potentially actionable. We refer to this group as “Actionable *PIK3CA*” (A).

We also retrieved data about rates of relapse-free survival related to 73/85 patients whose tumor was analyzed to devise the LAURA classification (remaining patients lost at follow-up). The “unique HER2-gain” category showed a higher rate of relapse-free survival; however, the difference with the other groups was not statistically significant (24% for U compared to 15% for A, 10% for LA, 8% for R). Regrettably, this retrospective non-consecutive cohort of HLBC patients does not allow to perform robust correlations with clinical data to appreciate whether a prognostic stratification of patients can be obtained. Indeed, there is an intrinsic heterogeneity of adjuvant treatments administered to patients (endocrine therapy only versus endocrine+chemo) across the groups, and the cases were collected over a wide period of time thus leading to different lengths of follow-up intervals.

## Discussion

Here, we show that HER2-low breast carcinomas harbor a constellation of somatic mutations that shows significant differences compared to HER2-positive and HER2-negative BCs. Nevertheless, significant differences were observed across distinct HER2-low subgroups (score 1+ versus score 2+) with *ERBB2* mutations uniquely observed in score 2+ carcinomas. Transcriptional profiling demonstrated a clear separation between score 1+ and score 2+ groups, with score 1+ carcinomas showing overlapping gene expression features with score 0 carcinomas and score 2+ with equivocal *HER2* gene copy numbers harboring the most distinct transcriptional profile. The latter group also harbored the highest *ERBB2* mRNA levels. Finally, a class discovery transcriptional classification highlighted the existence of four groups with distinct and unique features, also in terms of actionability.

The HLBCs included in the present study were composed of both ER-positive and ER-negative tumors, which were carefully characterized at the *ERBB2* gene level by FISH to provide a precise categorization of the heterogeneity of HLBCs that is already seen in clinical practice but never considered in translational studies (score 2+ carcinomas with normal *HER2* gene CN – HLBC-2N – or with *HER2* gene CN in the equivocal range – HLBC-2E). This fine refinement of the clinical categories enabled a precise contextualization of differences observed at the genomic level across the spectrum of HER2-low breast cancer.

In line with the luminal-B subtype prevalence, previously observed [9, 10] and here confirmed, the most frequently mutated genes were *PIK3CA* (33%), *GATA3* (18%), and *TP53* (17%), followed by *ERBB2* (8%). Interestingly, *TP53* mutations were found at a higher frequency in score 1+ carcinomas compared to the other groups, and *ERBB2* mutations were uniquely detected in the HLBC-2E subgroup. Score 1+ carcinomas also showed significantly higher TMB levels. This observation leads to the acknowledgment of potentially targetable alterations identified by applying a comprehensive genomic profiling allowing the investigation of TMB and MSI status. Indeed, our study provides an overview of the landscape of potentially targetable genetic alterations for HER2-low carcinomas by using a targeted approach that met 100% feasibility in FFPE tissue samples. By considering at least one alteration with an OncoKB [14]/ESCAT [15, 16] LoE, about half of the patients showed a potentially actionable alteration, including mainly *PIK3CA* and *ERBB2* mutations.

We next compared this genetic landscape with that of HER2-negative and HER2-positive diseases by using a publicly available MSKCC dataset that includes detailed information about HER2 scores and histopathological features. We performed multiple comparisons with cohorts that were both matched and not matched for histopathological and phenotypical features. The unmatched cohorts are informative to identify unique features of HLBCs per se. The matched cohorts are useful to create a comparison with BCs with analogous ER status and histological grade, which constitute two major determinants of their biology. Since we know that HLBCs are largely luminal carcinomas, the differences we identified may be at least partly driven by these features. HLBC-1 proved to be more similar to HER2-negative tumors, whereas HLBC score 2+ tumors had a mutational landscape that was superimposable to HER2-positive disease. Taken together, the genomic data stemming from the mutational analysis suggest that a separation within the family of HLBC exists between score 1+ (HLBC-1) and score 2+ (HLBC-2) carcinomas.

This separation was mirrored at the transcriptomic level. Even if HLBCs showed differences with both HER2-negative and HER2-positive carcinomas, intra-group analyses revealed overlapping features between score 0 and HLBC-1 cases, whereas score HLBC-2 showed an increasing differential gene expression that reached the highest levels of diversity for HLBC-2E carcinomas. This latter group showed an increased expression of the *ERBB2* signature, which was only partially retained in HLBC-2N tumors. HLBC-2E also showed an increase in *CDK4*, *PTEN*, and *RB1* signatures. Bao et al. [30] have recently investigated the association between low levels of *ERBB2* expression and clinical outcomes among patients with ER-positive/HER2-negative metastatic breast cancer patients treated with CDK4/6 inhibitors and described ERBB2-low expression as an independent predictor of inferior progression-free survival.

This observation is particularly interesting with respect to the novel perspective of the LAURA classification. The latter enables to appreciate the full picture of heterogeneity across HLBC, thus identifying distinctive features and allowing cross-correlation/integration with genetic alterations identified at DNA-based sequencing. The classification recognizes lymphocyte-activated tumors (both ER− and ER+), carcinomas with a unique enrichment in HER2-related features (higher *ERBB2* copy number, relative higher frequency of *ERBB2* mutations), carcinomas with remodeling of the tumor-stromal interface, and a class with the most pronounced actionability of *PIK3CA* mutations. We may hypothesize that the distinct features detected by our classification could be linked to the degree and quality of response to different therapeutic agents that are currently administered to HLBC patients. For instance, in the advanced setting, response rates to ADCs targeting HER2 are observed in about 30-50% of patients [4–6]. Furthermore, CDK4/6 inhibitors and *PIK3CA* inhibitors are standard of care for advanced luminal carcinomas, and as mentioned above, *ERBB2*-low expression has been recently reported as an independent predictor of inferior progression-free survival [30]. Data on *PIK3CA* mutational status were not available for further stratification in this study. Of note, the population eligible for *PIK3CA* inhibition [31] comprises a large portion of breast cancer patients with HER2-low disease. Whether the HER2-low status may affect response to *PIK3CA* inhibitors remains to be determined.

Our study has some limitations, the main being the lack of direct clinical relevance of the novel LAURA classification. This would need the application of the classification to homogeneously treated patient cohorts with the careful distinction of IHC scores. To the best of our knowledge, no cohorts as such are publicly available, and further studies will be needed in both early and advanced settings. In the present cohort, we retrieved data on rates of relapse-free survival, observing a higher, yet not significant, relapse rate in the U category. Regrettably, the retrospective nature of the non-consecutive cohort did not allow to perform robust correlations with the outcome, since patient treatments were heterogeneously distributed across categories, and there was a high variability of follow-up intervals. Further studies are warranted to investigate the potential prognostic and/or predictive refinement that this classification may provide in homogeneously treated patients.

Finally, one may question that a deeper molecular profiling beyond a targeted approach may unveil a more complete molecular underpinning of these tumors. Although this certainly holds true, our study provides a set of data that balances informativeness with the feasibility of comprehensive genomic profiling in archival FFPE samples, mirroring the approach adopted in diagnostic practice and clinical research when aiming to investigate carcinomas for possible rare molecular alterations and access to investigational drugs.

## Conclusions

Our integrative large-scale genomic and transcriptomic analysis disentangled the ontology of HLBCs providing evidence for the first time that (i) score 1+ and score 2+ carcinomas represent distinct entities and that (ii) four genomically distinct groups of tumors can be recognized, with possible implications in terms of stratification of response to selected anti-tumor agents. We could not provide direct evidence of the potential clinical impact of the novel LAURA classification; nevertheless, the data we report enable a better understanding of HER2-low disease and provide an overview of the potential treatment arsenal for HLBC patients that can guide treatment decision-making in clinical practice and a more accurate design of investigational clinical studies addressing HLBC.

## Supplementary Information

**Additional file 1.** Supplementary Methods.

**Additional file 2: Table S1.** Main pathological features of the HLBC-FPO Cohort.

**Additional file 3: Table S2.** Quality Controls (QCs) of TSO500.

**Additional file 4: Fig. S1.** Mutational signatures of HLBCs. The picture reports the real 96-matrix substitution identified in the whole cohort and in each HER2 class. We estimated the mutational signatures comprising all the variants with a VAF>0.1. Donut and bar plots show the relative representation of the best fitting COSMIC v2 signatures with the substitutions identified in the cohorts. **Fig. S2.** A. Comparison of Variant Classification (VC) distribution among HLBCs and control cohorts. B. Comparison of Variant Type (VT) distribution among HLBCs and control cohorts. C. Comparison of variant pathogenic levels among HLBCs and control cohorts. **Fig. S3.** Hematoxylin and Eosin (H&E) and immunohistochemical reaction with antibodies raised against MLH1 for HLBC-1-17 characterized by a high TMB (42.52 mut/Mb), microsatellite instability, lack of MLH1 expression. **Fig. S4.** Heatmaps of pathway scores for HLBCs. A. Unsupervised clustering of pathways score. Annotation for IHC-based classes does not reveal enriched pathways associated with the IHC subclassification. B-C-D. Heatmaps representing the genes belonging to cytokine and chemokines signaling (B), antigen presentation (C), stromal markers (D). Some cases show peculiar upregulation (or downregulation) in these gene sets, regardless of the IHC class. **Fig. S5.** NMF basic features for the LAURA Classification. A. Line plot of the cophenetic coefficient determined by the rank summary. Coefficients tending to 1 indicate the most robust clusters. A large decrease in the stability was detected for a cluster with 6 groups. Red circled dots represent the selected grouping strategy, the first being the achievement of at least four groups without superimposable features with the PAM50 subtypes. B. Consensus matrix for the four clusters. Annotation for IHC-Class, IHC-subtype, PAM50-subtype, basic genes for the cluster definition, consensus group and NMF silhouette is provided. C. Heatmap reporting the genes driving the clustering. **Fig. S6.** Genotypic and phenotypic features of the four clusters composing the LAURA Classification. A. Bar-plot describing the distribution of the PAM50 subtypes within the RNA classes. RNA classes are represented as colored dots. B. Bar-plot representing the tumor grade distribution within the RNA classes. C. Boxplot representing the Ki67 IHC score (continuous scale) within the RNA groups. D. Boxplot representing of TMB score (mut/Mb) within the RNA groups. E. OncoPrint of the 85 patients analyzed for class discovery. RNA-based classes are annotated in the bottom map. F. Dot plot depicting the ER and PgR expression values by IHC (continuous scale) within the RNA groups. **Fig. S7.** A. Boxplot representing the Adhesion and Migration score within the RNA groups. B. Boxplot representing the stromal markers score within the RNA groups. C. Representative micrographs of: i) a lobular carcinoma of classic type displaying a targetoid growth pattern around a residual duct and discohesiveness of tumor cells (see white spaces between cells), ii-iv) three different low grade carcinomas showing a marker fibro-elastotic stroma (arrows).

**Additional file 5: Table S3.** Germline variants in the HLBC-FPO Cohorts (Pathogenic and VUS).

**Additional file 6: Table S4.** P-values for mutation distribution comparison between HLBCs and MSKCC Cohorts.

**Additional file 7: Table S5.** Comparisons for VC and VT.

**Additional file 8: Table S6.** Variant pathogenicity annotation of the differentially mutated genes.

**Additional file 9: Table S7.** P-values for the CN gain comparison between HLBCs and MSKCC cohorts.

**Additional file 10: Table S8.** OncoKB/ESCAT annotation for all detected variants.

## Data Availability

All results are included in the main manuscript and in the additional material. The sequencing data related to the clinical samples are not disclosed as patients had not consented to the deposition of their sequence data.

## Abbreviations

ADCs: Antibody-drug conjugates
ASCO-CAP: American Association of Clinical Oncology – College of American Pathologist
BC: Breast cancer
ER: Estrogen receptor
ESCAT: ESMO Scale for Clinical Actionability of Molecular Targets
ESMO: European Society of Medical Oncology
DGE: Differential gene expression
FDR: False discovery rate
GSS: Global significance score
HLBC(s): HER2-low breast carcinoma(s)
IHC: Immunohistochemistry
ISH: In situ hybridization
MSI: Microsatellite instability
MSKCC: Memorial Sloan-Kettering Cancer Center
NMF: Non-negative matrix factorization
NST: Non-special histologic type
TILs: Tumor-infiltrating lymphocytes
TMB: Tumor mutational burden
TNBC: Triple-negative breast cancer
VC: Variant calling
VT: Variant type

## References

[1] Marchio C (2021). Evolving concepts in HER2 evaluation in breast cancer: heterogeneity, HER2-low carcinomas and beyond. Semin Cancer Biol.

[2] Allison KH (2020). Estrogen and progesterone receptor testing in breast cancer: ASCO/CAP guideline update. J Clin Oncol.

[3] Wolff AC (2018). Human epidermal growth factor receptor 2 testing in breast cancer: American Society of Clinical Oncology/College of American Pathologists Clinical Practice Guideline Focused Update. J Clin Oncol.

[4] Banerji U (2019). Trastuzumab duocarmazine in locally advanced and metastatic solid tumours and HER2-expressing breast cancer: a phase 1 dose-escalation and dose-expansion study. Lancet Oncol.

[5] Modi S (2020). Antitumor activity and safety of trastuzumab deruxtecan in patients with HER2-low-expressing advanced breast cancer: results from a phase Ib study. J Clin Oncol.

[6] Modi S (2022). Trastuzumab deruxtecan in previously treated HER2-low advanced breast cancer. N Engl J Med.

[7] Tarantino P (2020). HER2-low breast cancer: pathological and clinical landscape. J Clin Oncol.

[8] Agostinetto E (2021). HER2-low breast cancer: molecular characteristics and prognosis. Cancers (Basel).

[9] Schettini F (2021). Clinical, pathological, and PAM50 gene expression features of HER2-low breast cancer. NPJ Breast Cancer.

[10] Marchio C (2018). The dilemma of HER2 double-equivocal breast carcinomas: genomic profiling and implications for treatment. Am J Surg Pathol.

[11] N.I.H. US National Library of Medicine ClinicalTrial.govhttps://clinicaltrials.gov/ct2/show/NCT03347318?term=candiolo+profiling&draw=2&rank=1 10.1080/15360280801989377 28792816

[12] Razavi P (2018). The genomic landscape of endocrine-resistant advanced breast cancers. Cancer Cell.

[13] Breast cancer (MSK, Cancer Cell 2018) https://www.cbioportal.org/study/summary?id=breast_msk_2018

[14] Chakravarty D, et al. OncoKB: a precision oncology knowledge base. JCO Precis Oncol. 2017;1:PO.17.00011. 10.1200/PO.17.00011. Published online 2017 May 16. 10.1200/PO.17.00011 PMC5586540 28890946

[15] Condorelli R (2019). Genomic alterations in breast cancer: level of evidence for actionability according to ESMO Scale for Clinical Actionability of molecular Targets (ESCAT). Ann Oncol.

[16] Mateo J (2018). A framework to rank genomic alterations as targets for cancer precision medicine: the ESMO Scale for Clinical Actionability of molecular Targets (ESCAT). Ann Oncol.

[17] Berrino E (2022). Collision of germline POLE and PMS2 variants in a young patient treated with immune checkpoint inhibitors. NPJ Precis Oncol.

[18] Li Q, Wang K (2017). InterVar: clinical interpretation of genetic variants by the 2015 ACMG-AMP guidelines. Am J Hum Genet.

[19] Landrum MJ (2018). ClinVar: improving access to variant interpretations and supporting evidence. Nucleic Acids Res.

[20] Wang J (2021). Disease spectrum of breast cancer susceptibility genes. Front Oncol.

[21] Wang K, Li M, Hakonarson H (2010). ANNOVAR: functional annotation of genetic variants from high-throughput sequencing data. Nucleic Acids Res.

[22] Danaher P (2017). Gene expression markers of tumor infiltrating leukocytes. J Immunother Cancer.

[23] Gaujoux R, Seoighe C (2010). A flexible R package for nonnegative matrix factorization. BMC Bioinformatics.

[24] Parker JS (2009). Supervised risk predictor of breast cancer based on intrinsic subtypes. J Clin Oncol.

[25] Alexandrov LB (2020). The repertoire of mutational signatures in human cancer. Nature.

[26] Alexandrov LB (2013). Signatures of mutational processes in human cancer. Nature.

[27] Kauraniemi P, Kallioniemi A (2006). Activation of multiple cancer-associated genes at the ERBB2 amplicon in breast cancer. Endocr Relat Cancer.

[28] Yang Y (2021). Functional cooperation between co-amplified genes promotes aggressive phenotypes of HER2-positive breast cancer. Cell Rep.

[29] Patel A (2022). Gene-level germline contributions to clinical risk of recurrence scores in Black and White patients with breast cancer. Cancer Res.

[30] Bao KKH, Sutanto L, Tse SSW, Man Cheung K, Chan JCH (2021). The association of ERBB2-low expression with the efficacy of cyclin-dependent kinase 4/6 inhibitor in hormone receptor-positive, ERBB2-negative metastatic breast cancer. JAMA Netw Open.

[31] Andre F (2021). Alpelisib plus fulvestrant for PIK3CA-mutated, hormone receptor-positive, human epidermal growth factor receptor-2-negative advanced breast cancer: final overall survival results from SOLAR-1. Ann Oncol.

