# Supplementary material for: Integrative genomic and transcriptomic analyses illuminate the ontology of HER2-low breast carcinomas
Source: Genome Med. 2022 Aug 29;14:98. doi: 10.1186/s13073-022-01104-z (PMC9426037; doi:10.1186/s13073-022-01104-z)
Supplement: Supplementary file 1 — Additional file 1. Supplementary Methods. [file 13073_2022_1104_MOESM1_ESM.docx]

**ADDITIONAL FILE 1: SUPPLEMENTARY METHODS**

**Cohorts and related analyses**

We selected 99 breast carcinoma patients lacking *ERBB2* amplification (*ERBB2*/CEP17 ratio <2, *ERBB2* copy numbers <6 by FISH analysis) and showing either a score 1+ or 2+ in IHC. This represented the HER2-low Breast Cancer FPO-IRCCs Cohort (HLBC-FPO). IHC stainings (4B5 assay by Roche Ventana, Tucson, USA) were re-assessed by two pathologists (CM, AS) based on the latest ASCO-CAP guidelines[1] and the final cohort was subdivided into three IHC-based categories: 34 score 1+ (HLBC-1), 15 score 2+ with *HER2* gene copy numbers <4 (HLBC-2N), 50 score 2+ with *HER2* gene copy numbers between 4 and 6 (HLBC-2E). This is a series of non-consecutive cases, selected based on optimal tissue fixation (to avoid possible biases in the definition of HER2-low category), more than 90% of tumor cell content after mesodissection [2] (to allow downstream analysis) and availability of written informed consent (“Profiling” protocol approved by the Ethical committee of Candiolo Cancer Institute). Immuno-stainings for ER (Clone EP1, Dako, Agilent Technologies, Santa Clara, California, US), PgR (Clone 636, Dako, Agilent Technologies, Santa Clara, California, US) and Ki67 (Clone MIB1, Dako, Agilent Technologies, Santa Clara, California, US) were performed on a Leica Bond Autostainer (Leica microsystems).

The study includes two sets of control cohorts. To compare the mutational landscape of HLBC-FPO with HER2-negative and HER2-positive carcinomas publicly data were exploited since studies with targeted sequencing approaches are available (MSKCC cohort [3], see section below and Consort diagram in Figure 1B).

Conversely, since for gene expression analysis we used a NanoString approach that has not been widely reported by others we profiled a series of 30 HER2-negative and 15 HER2-positive breast carcinomas (see section below and Figure 1C).

**DNA extraction and targeted sequencing**

Genomic DNA was extracted from formalin-fixed paraffin-embedded (FFPE) tissue blocks of the 99 samples, following review of hematoxylin and eosin (H&E) to assess tumor cellularity and to identify tumor areas to be mesodissected. When needed, microdissection based on H&E or HER2-stained sections was performed under a stereomicroscope [2].

DNA was extracted using the GeneRead DNA FFPE Kit (Qiagen, Hilden, Germany) and quantified with spectrophotometric (Nanodrop 1000, ThermoFisher Scientific, Waltham, MA, US) and fluorometric (Qubit, ThermoFisher Scientific, Waltham, MA, US) assays. Eighty nanograms of genomic DNA were subjected to deep sequencing using the TruSight Oncology 500 (TSO500) panel (Illumina, San Diego, California, U.S). The targeted panel is 1.94 Mb in size, encompassing the exon sequence of 523 genes, mostly cancer-related (coding size 1.2 Mb). The panel allows the assessment of microsatellite instability (MSI) status (approximately 120 loci), tumor mutation burden (TMB), and copy number (CN) data (about 59 genes), following the manufacturer's protocol. Libraries were sequenced on a NovaSeq 6000 instrument (Illumina, San Diego, California, USA) to reach a minimum of 500X read depth.

Raw data were processed by the Illumina Local App associated with the TSO500 panel (TruSight Oncology 500 v2.2 Local App) to produce .fastq files through the alignment of the sequence to the human reference sequence GRCh37 (hg19). The Local App also performed sequencing QCs (Reported in Supplementary Table S2) and somatic variant calling with a tumor-only pipeline. The TMB was assessed as the ratio between the total somatic, non-synonymous variants with a (Variant Allele Frequency) VAF > 0.05 and the real sequence genome for each sample. We considered as confident all the somatic, non-synonymous SNVs and small indels with a VAF > 0.1. As for copy number (CN) analysis, we defined as numerically gain or loss genes with a Fold-Change > 4 and < 0.5, respectively. TMB among groups distribution data were assessed by using Unpaired T-test, two-tailed.

OncoPrint for data visualization were produced using the OncoPrint function of the ComplexHeatmap R package [4]. FC heatmap for CNA was built using the heatmap2 function of the ggplo2 R package. Significantly differentially mutated or CNA genes were evaluated by applying multiple Fisher’s and Unpaired t-test, two-tailed, with false discovery rate (FDR) adjustment, respectively.

Data about actionability were annotated using the OncoKB annotation [5], performed using the command line python tool oncokb/oncokb-annotator, and with the ESMO Scale for Clinical Actionability of Molecular Targets (ESCAT) [6, 7]. The related lollipop plots were produced using the g3viz R package. We reported all the mutations affecting a gene with an OncoKB annotation of “oncologic” significance, and then we annotated the OncoKB/ESCAT Level of Evidence (LoE). CN alterations (CNAs) were annotated by following GISTIC 2.0 format, as imposed by the python tool oncokb/oncokb-annotator.

The mutational signatures were evaluated using the six substitution subtypes: C>A, C>G, C>T, T>A, T>C, and T>G with a VAF > 0.1. Mutalisk tools [8] was applied to calculate the most prevalent signatures for each HER2-low group and subgroup, by comparing sample signature with the ones reported in the Catalogue of Somatic Mutations in Cancer (COSMIC) database v. 214.

InterVar tool [9] was applied to annotate the TSO500, germline-filtered variants.

ClinVar significance [10] was used to define the pathogenic and the Variants with Unknown Significance (VUS) variants in the present cohort.

The pathogenicity level was use by annotating with 12 different prediction tools belonging to the filter-based pipeline of ANNOVAR [11]. We grouped the variants in 4 pathogenic groups: nonsense (frameshift or stopping codon variants), damaging (with at least 9 tools classifying as damaging), potentially pathogenic (with 6-8 tools classifying as damaging) and tolerated (less than 6 tools classifying as damaging).

**RNA extraction and targeted gene expression profiling**

RNA was extracted with the High Pure FFPET RNA Isolation Kit (Roche, Basel, Switzerland) from the 91 HLBCs and from 30 HER2-negative and 15 HER2-positive patients. RNA samples were quantified with both spectrophotometric (Nanodrop 1000) and fluorometric (Qubit) assays; the integrity was assessed by the BioAnalyzer 6000 Nano Assay on the BioAnalyzer 2100 instrument (Agilent, Santa Clara, CE, US). Targeted gene expression profiling (tGEP) was carried out by using the BreastCancer BC 360™ Panel (NanoString Technologies, Seattle, WA, US), comprising 776 BC related genes following the manufacturer's protocol. The nSolver Software (NanoString Technologies) with the nSolver Advanced Analyses, based on robust R statistics, performed a primary level analysis with the pathway score calculated as the first principal component of the pathway genes normalized expression, the cell type score (method described in [12]) and the differential gene expression (DGE) analysis, where genes are tested for differential expression in response to each covariate (ER status, IHC-subtype and tumor grade) and grouped using hierarchical clustering with Euclidean distance. For the DGE analysis, a single linear regression is fit using all selected covariates to predict expression for each gene. This approach eliminates confounding due to measured covariates and isolates the independent association of each covariate with gene expression, measuring each variable's association with a gene after holding all other variables constant. Based on this, we evaluated: i) inter-group tGEP analysis comparing the expression levels of HLBCs vs the control groups; ii) inter-group tGEP analysis comparing the expression levels of IHC-based category of the HER2-low tumors. To confront HLBCs to the control groups we summarized the gene expression to gene set level, using the nSolver calculated global significance score (GSS) and the direct global significance score (dGSS). The GSS represented the overall differential expression of gene sets, independently from the up- or down-regulation, whereas the dGSS represented the gene set up and down regulated. All the Algorithm details were described in the nCounter Advanced Analysis 2.0 User Manual (MAN-10030-03). A private NanoString algorithm performed secondary evaluation, comprising the PAM50 intrinsic molecular subtyping and the Genomic Risk Score [13, 14]. On the tGEP data we also applied a consensus based non-negative matrix factorization (NMF) approach to perform class discovery using the R tools NMF [15]. We selected the most variable genes (top 33% genes) and NMF was carried out by using predetermined number of clusters (K), which varied from 2 to 6. We selected the clustering size by i) ranking the best cophenetic coefficients, ii) discarding clustering overlapping the PAM50 intrinsic subtyping, to identify new RNA-based classes. After class discovery, nSolver Advanced Analysis were re-applied to evaluate differential gene expression analysis between the classes.

**Assembly of the control cohort for comparison of mutational landscapes**

To compare mutational data with a control cohort, we screened all the BC open datasets of gene mutations available on cBioportal [16]. To allow a balanced comparison, the control set needed to contain: i) score-based evaluation of HER2 IHC data for the HER2-low group and subgrouping definition; ii) mutational data generated from a superimposable DNA based targeted panel to compare similar wet and bioinformatic conditions; iii) clinico-pathological data to match the features of the HLBC-FPO cohort (at least information about ER status and histological grade).

Taking into account the previous parameters, the MSKCC Breast Cancer [3] cohort resulted the best available control cohort. All the mutational and clinical data were downloaded the results from [17]. From a total of 1756 samples, we derived two different comparative population: i) the Unmatched Cohort, comprising 1317 samples with all mandatory fields described in point i and ii of the cohort selection, but without the “normalization” with the HLBC-FPO cohort; ii) the Matched Cohort, composed of 545/1317 samples, in which we selected the number of HER2-negative, 1+, 2+ and 3+ samples of the MSKCC-cohort taking into account the prevalence of ER status and the tumor grade of the HLBC-FPO cohort. In details, each *HER2* group of the control cohort must be composed by 90% of ER-positive and 10% of ER-negative samples. Within these ER classes, we also matched the tumor grade, with the ER-positive samples comprising 55% of high-grade tumors (G3) and with the ER-negative samples composed by the 80% of G3 tumors. The final MSKCC cohorts are summarized in the flow-chart of Figure 1B.

Mutational data of the control cohorts were downloaded and intersected with the TSO500 manifest files to selected only the shared genes. To statistically assess the differential mutation landscape, we compare the number of mutations for each gene of the HLBC-FPO cohort with the HER2-negative, the HER2-low and the HER2 score 3+ groups of the MSKCC unmatched and matched cohorts. Moreover, we also compared the number of mutations between the IHC-based category within the HLBC-FPO population. To do this, we applied multiple Fisher’s tests, two-tailed, with R custom script, and calculated both the unadjusted and FDR-adjusted *P-*value. We calculated both the *P-*value (significance < 0.01) and the *P-*value adjusted with FDR (significance < 0.05), to define the genes with a differential prevalence between the groups. For comparisons involving the IHC-based subdivision of the HLBC-FPO cohort, the FDR adjusted *P-*value was less informative than the standard *P-*value, due to the relatively low number of mutations and of samples/category. The radar plots were built with the fmsb R package. We also evaluated the different distribution of CN gain between the cohort, comparing the number of positive patients with CNAs for each gene comprised in the TSO500 panel by applying the same Fisher Exact test, twotailed, for the previously defined cohorts.

**FISH analysis**

FISH analysis was performed for copy number validation assay on interphase cells using 4 µm-thick sections of FFPE samples. For this purpose, the following probes were adopted: ZytoLight ® SPEC KRAS/CEN 12 Dual Color Probe, ZytoLight ® SPEC MET/CEN 7 Dual Color Probe, ZytoLight ® CEN 17/SPEC ERBB2 Dual Color Probe and ZytoLight ® SPEC FGFR1/CEN 8 Dual Color Probe (Zytovision, Bremerhaven, Germany) following the manufacturer’s instructions.

Briefly, FFPE sections were deparaffinized, air-dried, incubated in Pre-Treatment Solution at 98 °C for 10 min, followed by proteolytic digestion using Pepsin (Agilent, Santa Clara, United States). After air dehydration, 10 μl of probe mixture were applied to each sample. Slides and probes were co-denatured at 75 °C for 10 min and hybridized at 37 °C for 16 h in the dark (Top Brite, Resnova S.r.l., Roma, Italy). After washing with Stringent Wash Buffer (Agilent, Santa Clara, United States), chromatin was counterstained with DAPI 150 ng/ml (Zytovision, Bremerhaven, Germany). An average of fifty cells were analyzed using an Olympus BX61 microscope (Olympus Corporation, Tokyo, Japan) and analyzed by CytoVision software 7.2 (Leica Biosystems, Newcastle Ltd., UK).

*KRAS*, *MET*, *FGFR1, CCND1* and *HER2* status was defined by calculating the Ratio (R) between the gene and the centromere probe copy number and considered amplified if R ≥ 2.0.

**Confirmatory MSI analysis**

MMR protein expression was tested using 4 µm-thick sections cut from FFPE blocks and subjected to IHC with antibodies raised against MLH1 (M1, Roche-Ventana, Oro Valley, AZ, US), anti-MSH2 (G219-1129, Roche-Ventana), anti-MSH6 (SP-93, Roche-Ventana) and anti-PMS2 (A16-4, Roche-Ventana) using the Roche BenchMark ULTRA instrument (Roche-Ventana).

Molecular confirmation of instability was assessed by using the MSI analysis system - version 1.2 (Promega, Madison, WI, US), which includes five mononuclear and monomorphic microsatellite loci following the manufacturer’s protocol. Products were analyzed by capillary electrophoresis using an ABI 3100 Genetic Analyzer (Applied Biosystems, Foster City, CA, US).

**Immunohistochemistry for PD-L1 expression**

Those cases pertaining to the ‘Lymphocyte Activated’ (LA) transcriptomic group were subjected to PD-L1 assays.

Briefly, hree micron thick FFPE tissue sections were stained with the SP142 assay (Roche-Ventana) on the Roche BenchMark ULTRA instrument (Roche-Ventana) and with the 22C3 assay (Dako Agilent) on the Dako Autostainer (Dako Agilent), following the manufacturers’ instructions.

PD-L1 scoring was assessed based on assay-specific recommendations, i.e. the Immune Cell (IC) scoring for the SP142 assay [18] and the Combined Positive Score (CPS) for the 22C3 [19].

**REFERENCES**

1. Wolff AC, et al: **Human Epidermal Growth Factor Receptor 2 Testing in Breast Cancer: American Society of Clinical Oncology/College of American Pathologists Clinical Practice Guideline Focused Update.** *J Clin Oncol* 2018, **36:**2105-2122.

2. Marchio C, et al: **The genetic landscape of breast carcinomas with neuroendocrine differentiation.** *J Pathol* 2017, **241:**405-419.

3. Razavi P, et al: **The Genomic Landscape of Endocrine-Resistant Advanced Breast Cancers.** *Cancer Cell* 2018, **34:**427-438 e426.

4. Gu Z, Eils R, Schlesner M: **Complex heatmaps reveal patterns and correlations in multidimensional genomic data.** *Bioinformatics* 2016, **32:**2847-2849.

5. Chakravarty D, et al: **OncoKB: A Precision Oncology Knowledge Base.** *JCO Precis Oncol* 2017, **2017**.

6. Condorelli R, et al: **Genomic alterations in breast cancer: level of evidence for actionability according to ESMO Scale for Clinical Actionability of molecular Targets (ESCAT).** *Ann Oncol* 2019, **30:**365-373.

7. Mateo J, et al: **A framework to rank genomic alterations as targets for cancer precision medicine: the ESMO Scale for Clinical Actionability of molecular Targets (ESCAT).** *Ann Oncol* 2018, **29:**1895-1902.

8. Lee J, et al: **Mutalisk: a web-based somatic MUTation AnaLyIS toolKit for genomic, transcriptional and epigenomic signatures.** *Nucleic Acids Res* 2018, **46:**W102-W108.

9. Li Q, Wang K: **InterVar: Clinical Interpretation of Genetic Variants by the 2015 ACMG-AMP Guidelines.** *Am J Hum Genet* 2017, **100:**267-280.

10. Landrum MJ, et al: **ClinVar: improving access to variant interpretations and supporting evidence.** *Nucleic Acids Res* 2018, **46:**D1062-D1067.

11. Wang K, Li M, Hakonarson H: **ANNOVAR: functional annotation of genetic variants from high-throughput sequencing data.** *Nucleic Acids Res* 2010, **38:**e164.

12. Danaher P, et al: **Gene expression markers of Tumor Infiltrating Leukocytes.** *J Immunother Cancer* 2017, **5:**18.

13. Parker JS, et al: **Supervised risk predictor of breast cancer based on intrinsic subtypes.** *J Clin Oncol* 2009, **27:**1160-1167.

14. Patel A, et al: **Gene-Level Germline Contributions to Clinical Risk of Recurrence Scores in Black and White Patients with Breast Cancer.** *Cancer Res* 2022, **82:**25-35.

15. Gaujoux R, Seoighe C: **A flexible R package for nonnegative matrix factorization.** *BMC Bioinformatics* 2010, **11:**367.

16. **Breast Cancer (MSK, Cancer Cell 2018)**

17. **Breast Cancer (MSK, Cancer Cell 2018)**

18. Schmid P, et al: **Atezolizumab and Nab-Paclitaxel in Advanced Triple-Negative Breast Cancer.** *N Engl J Med* 2018, **379:**2108-2121.

19. Cortes J, et al: **Pembrolizumab plus chemotherapy versus placebo plus chemotherapy for previously untreated locally recurrent inoperable or metastatic triple-negative breast cancer (KEYNOTE-355): a randomised, placebo-controlled, double-blind, phase 3 clinical trial.** *Lancet* 2020, **396:**1817-1828.
