# Supplementary material for: Integrative genomic and transcriptomic analyses illuminate the ontology of HER2-low breast carcinomas
Source: Genome Med. 2022 Aug 29;14:98. doi: 10.1186/s13073-022-01104-z (PMC9426037; doi:10.1186/s13073-022-01104-z)
Supplement: Supplementary file 2 — Additional file 2: Table S1. Main pathological features of the HLBC-FPO Cohort. [file 13073_2022_1104_MOESM2_ESM.docx]

| Table S1. Main pathological features of the HLBC-FPO Cohort | | | | | |
| --- | --- | --- | --- | --- | --- |
|  |  | | # | % |  |
| Hystology | NST | 81 | | 82.0 |  |
|  | Lobular | 7 | | 7.0 |  |
|  | Mixed | 7 | | 7.0 |  |
|  | Other (Special type) | 4 | | 4.0 |  |
| ER status | Positive | 88 | | 88.9 |  |
|  | Negative | | 11 | 11.1 |  |
| Tumor Grade | G1-2 | | 43 | 43.0 |  |
|  | G3 | | 56 | 57.0 |  |
| Ki67 | <20% | | 32 | 32.3 |  |
|  | >20% | | 67 | 67.7 |  |
| pT | 1 | | 38 | 38.4 |  |
|  | 2 | | 57 | 57.6 |  |
|  | 3 | | 3 | 3.0 |  |
|  | 4 | | 1 | 1.0 |  |
| pN | X | | 3 | 3.0 |  |
|  | 0 | | 45 | 45.5 |  |
|  | 1 | | 37 | 37.4 |  |
|  | 2 | | 8 | 8.1 |  |
|  | 3 | | 6 | 6.0 |  |
| IHC Subtype | Luminal A | | 9 | 9.1 |  |
|  | Luminal B | | 81 | 81.8 |  |
|  | TNBC | | 9 | 9.1 |  |
| PAM50 Subtype | LumA | | 29 | 29.3 |  |
|  | LumB | | 50 | 50.5 |  |
|  | HER2-E | | 4 | 4.1 |  |
|  | Basal | | 6 | 6.1 |  |
| Age at diagnosis | Mean (CI95%) | |  |  |  |
| (years) | 62 (59-66) | |  |  |  |
| Size | Mean (CI95%) | |  |  |  |
|  | 25 (21-27) | |  |  |  |
