# Supplementary figures and images for: Integrative genomic and transcriptomic analyses illuminate the ontology of HER2-low breast carcinomas
Source: Genome Med. 2022 Aug 29;14:98. doi: 10.1186/s13073-022-01104-z (PMC9426037; doi:10.1186/s13073-022-01104-z)

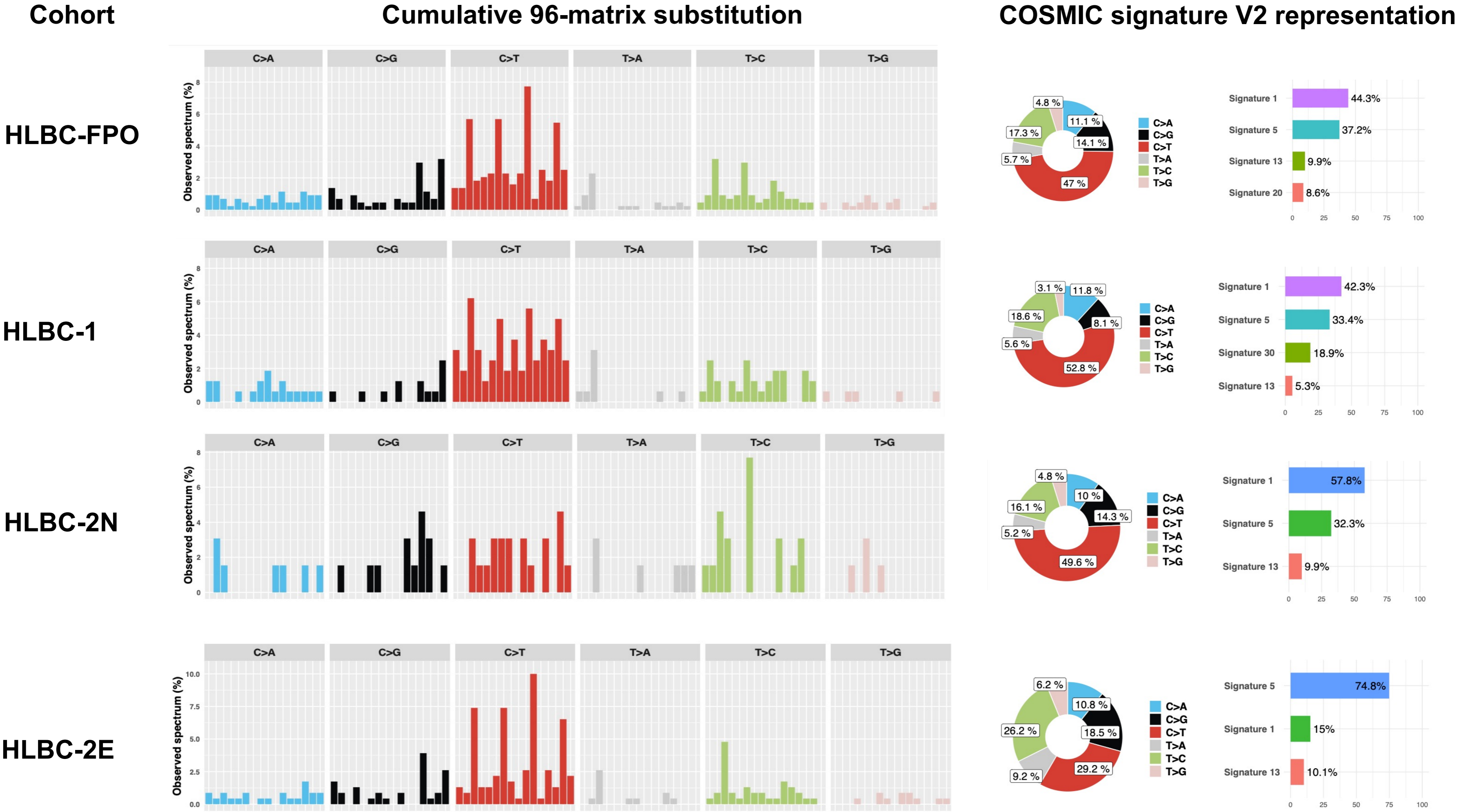

Fig. S1

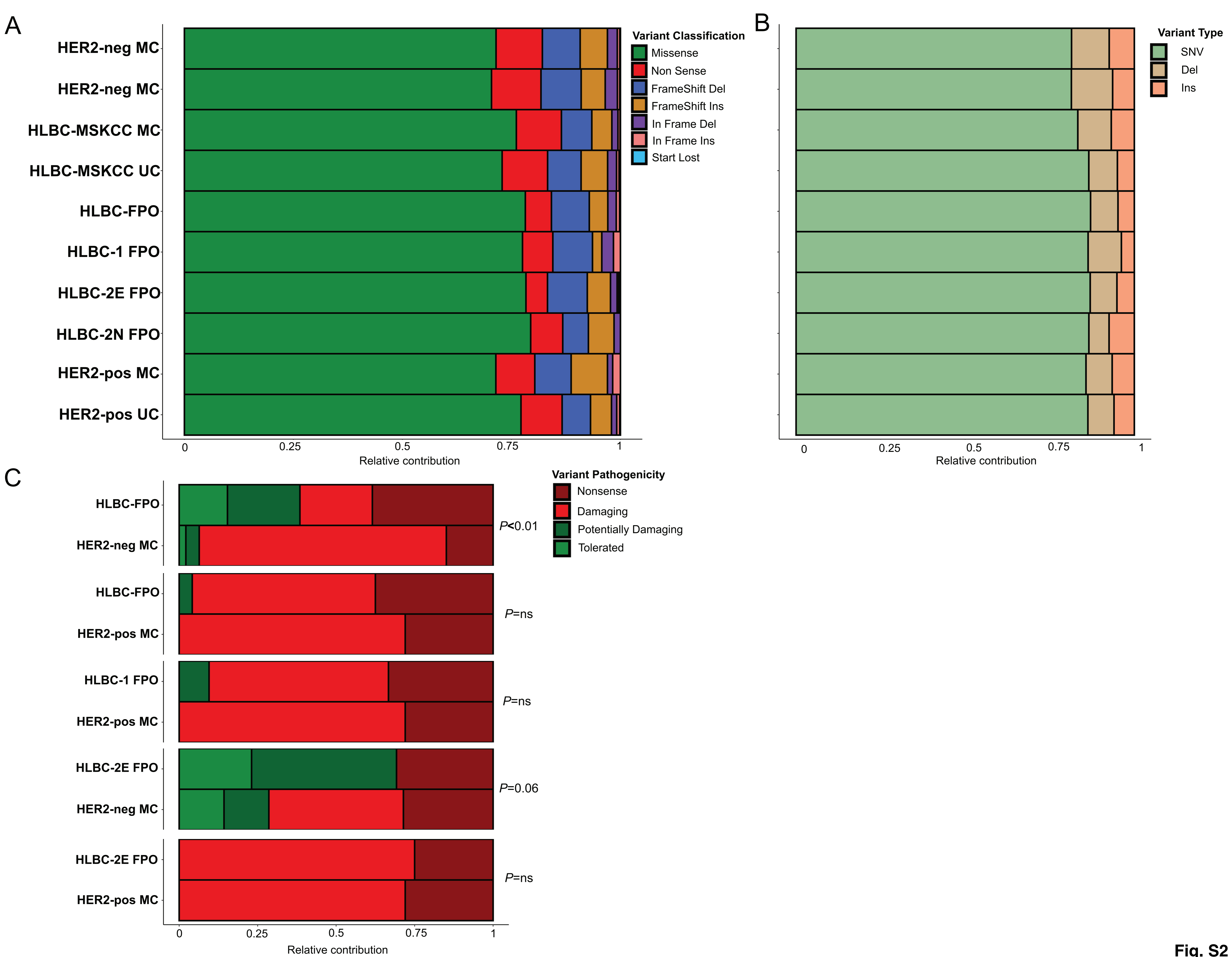

Fig. S2

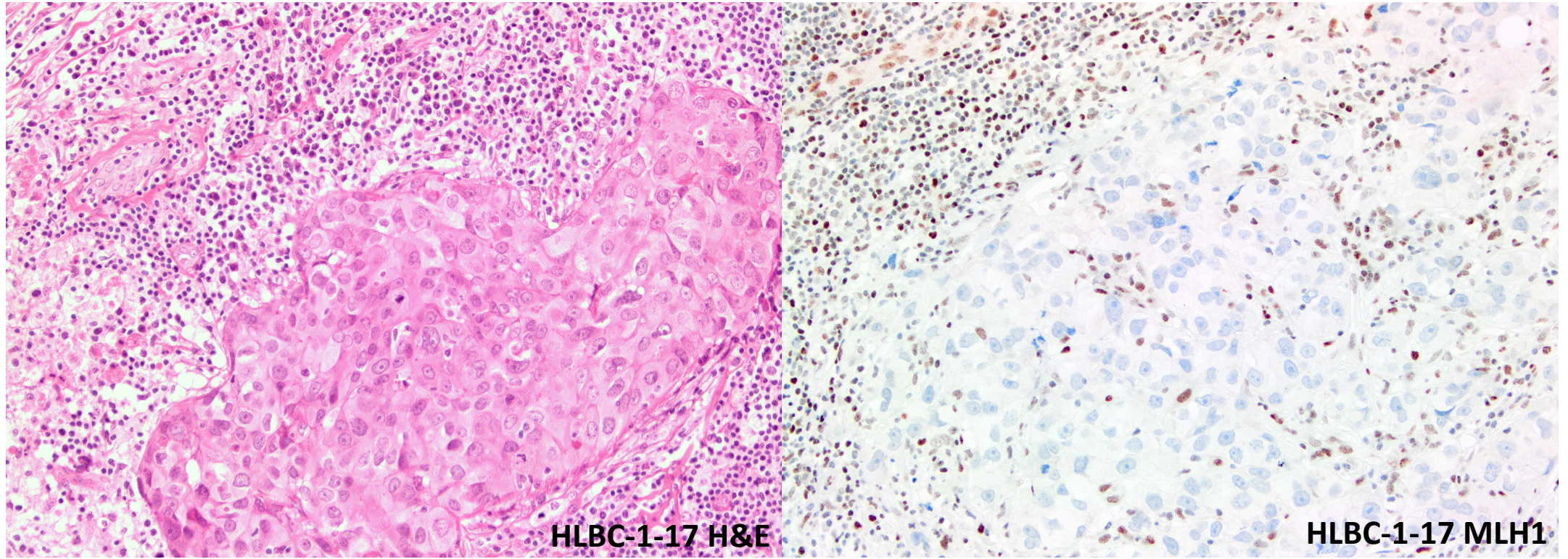

**Fig. S3**

A

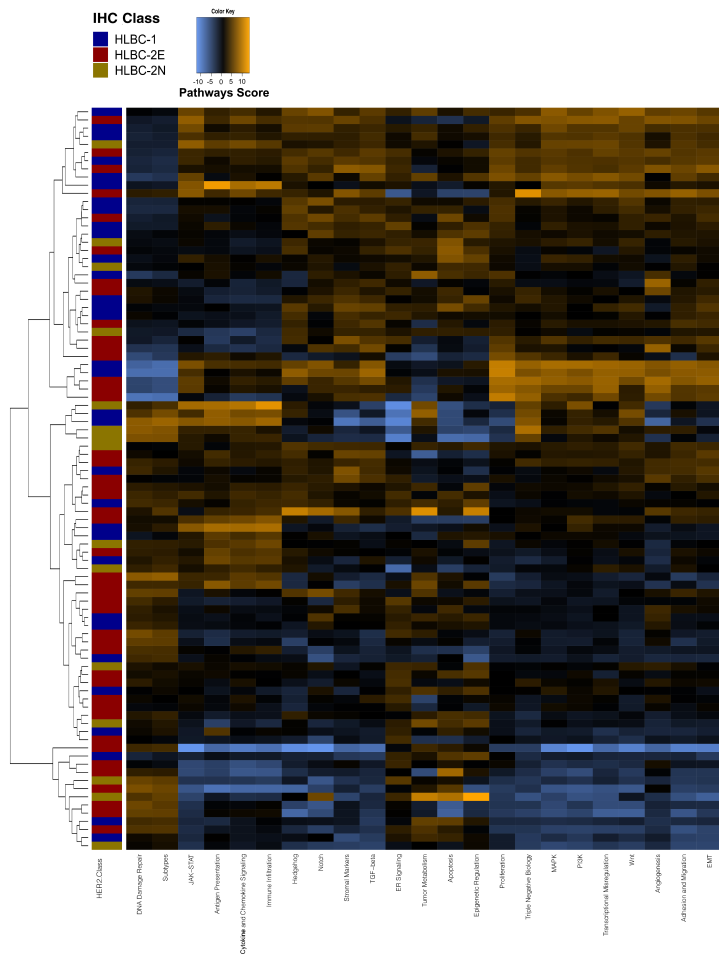

B

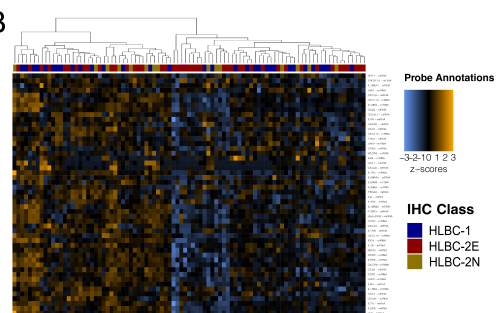

C

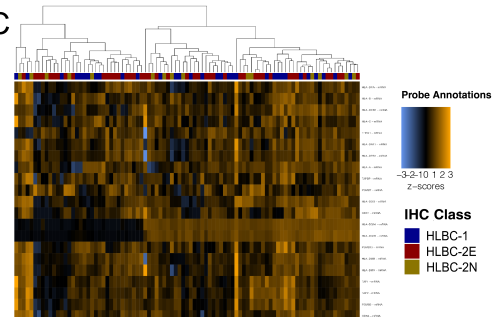

D

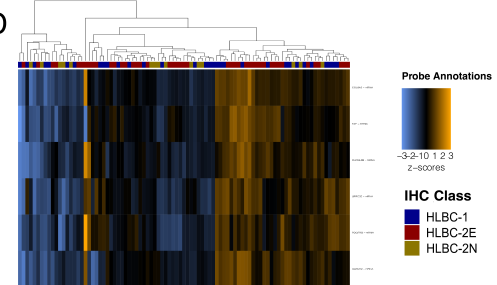

Fig. S4

A

## NMF rank survey

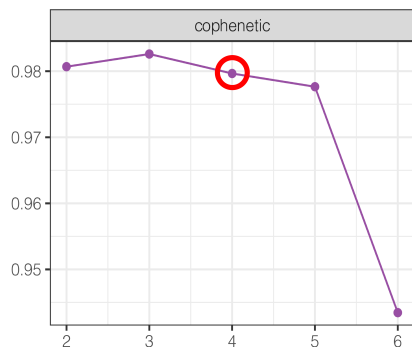

B

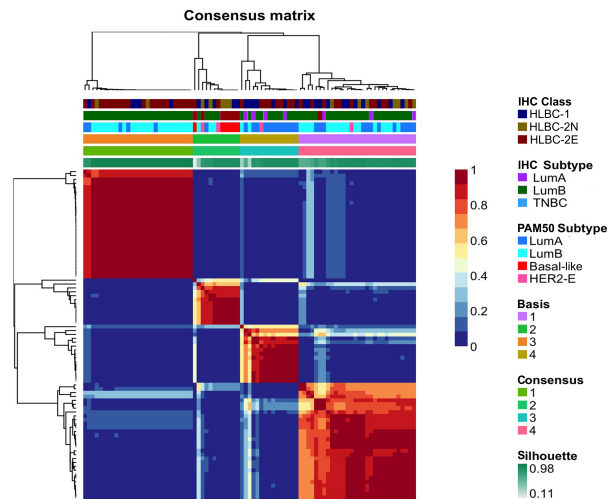

C

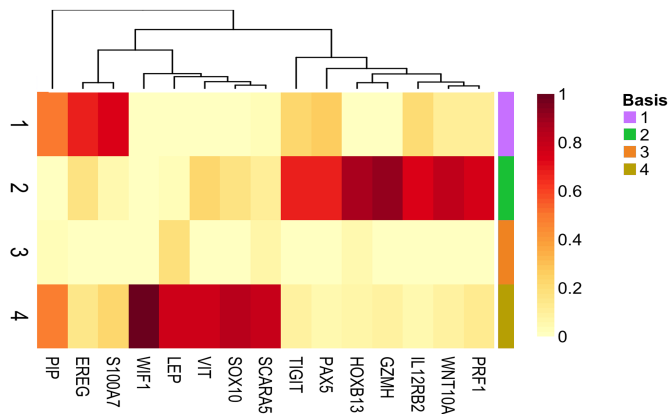

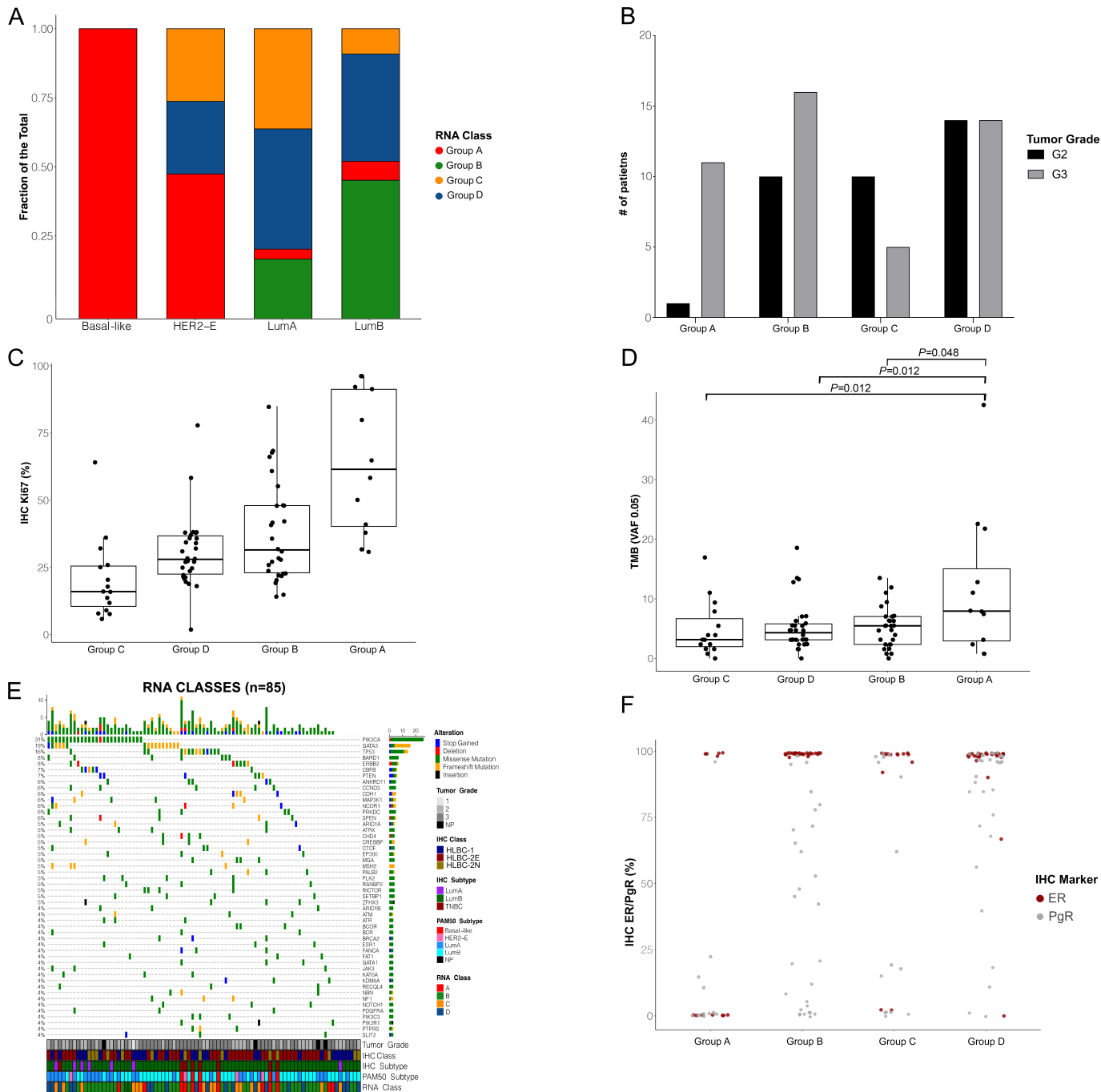

Fig.S6

A

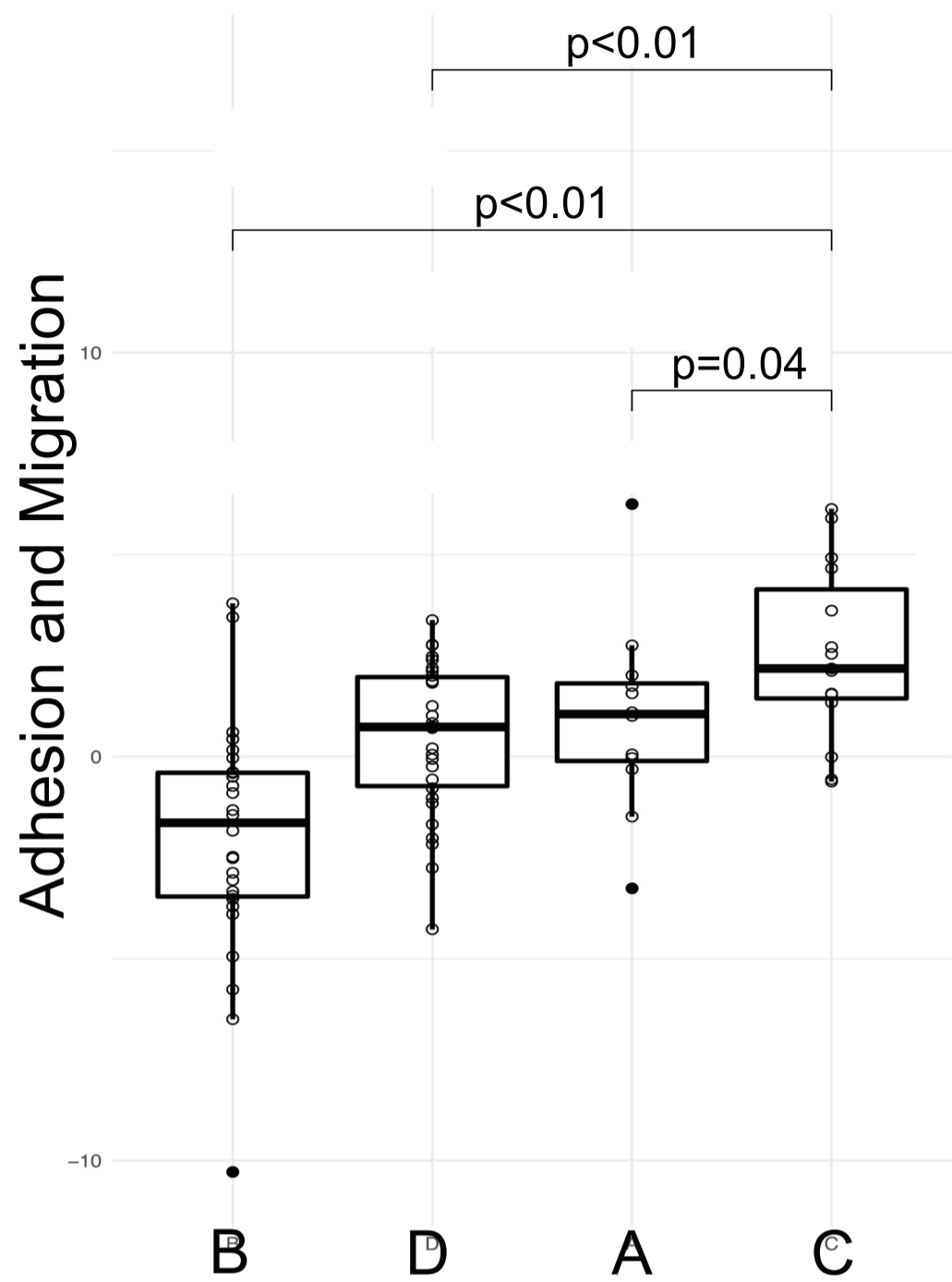

B

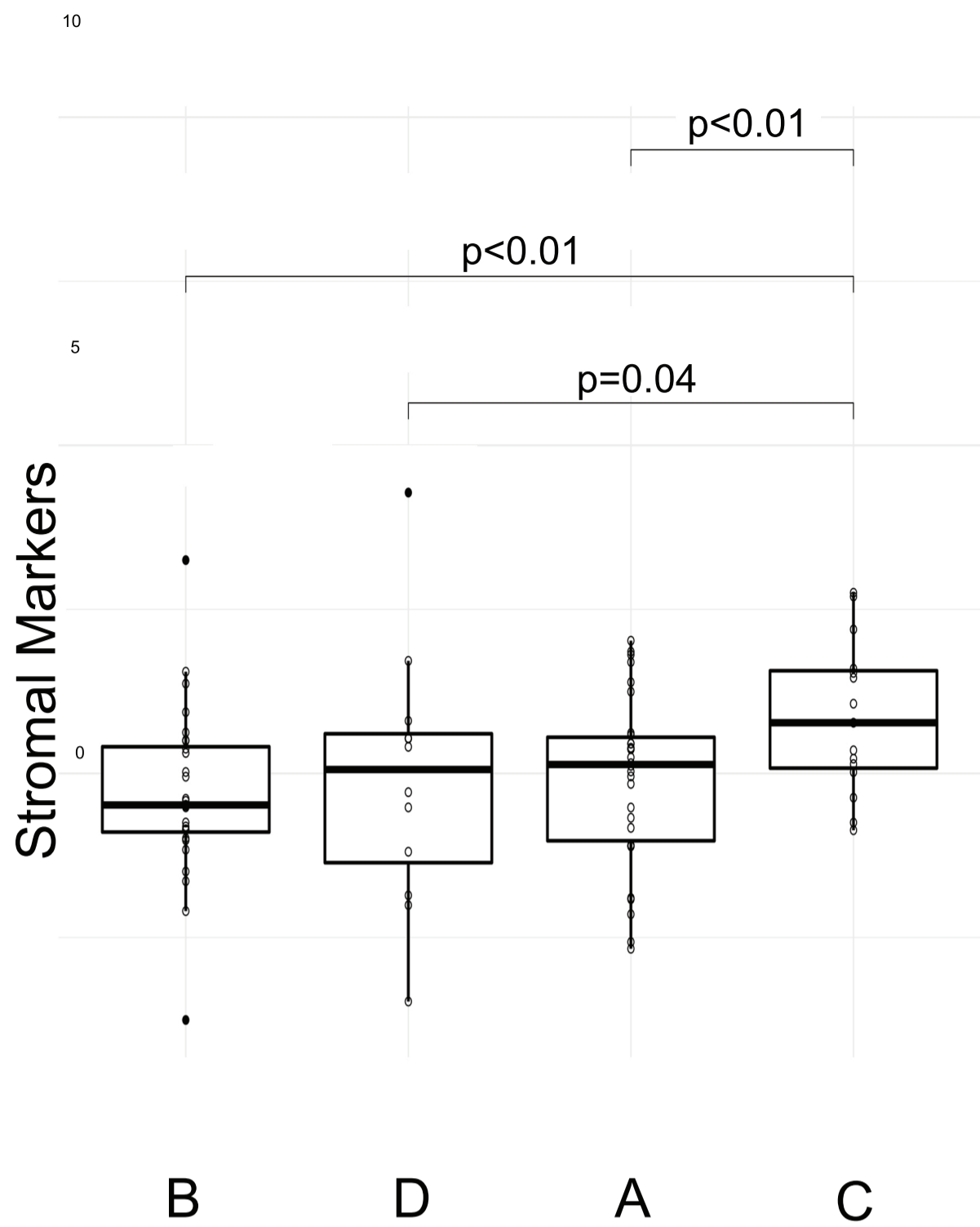

C

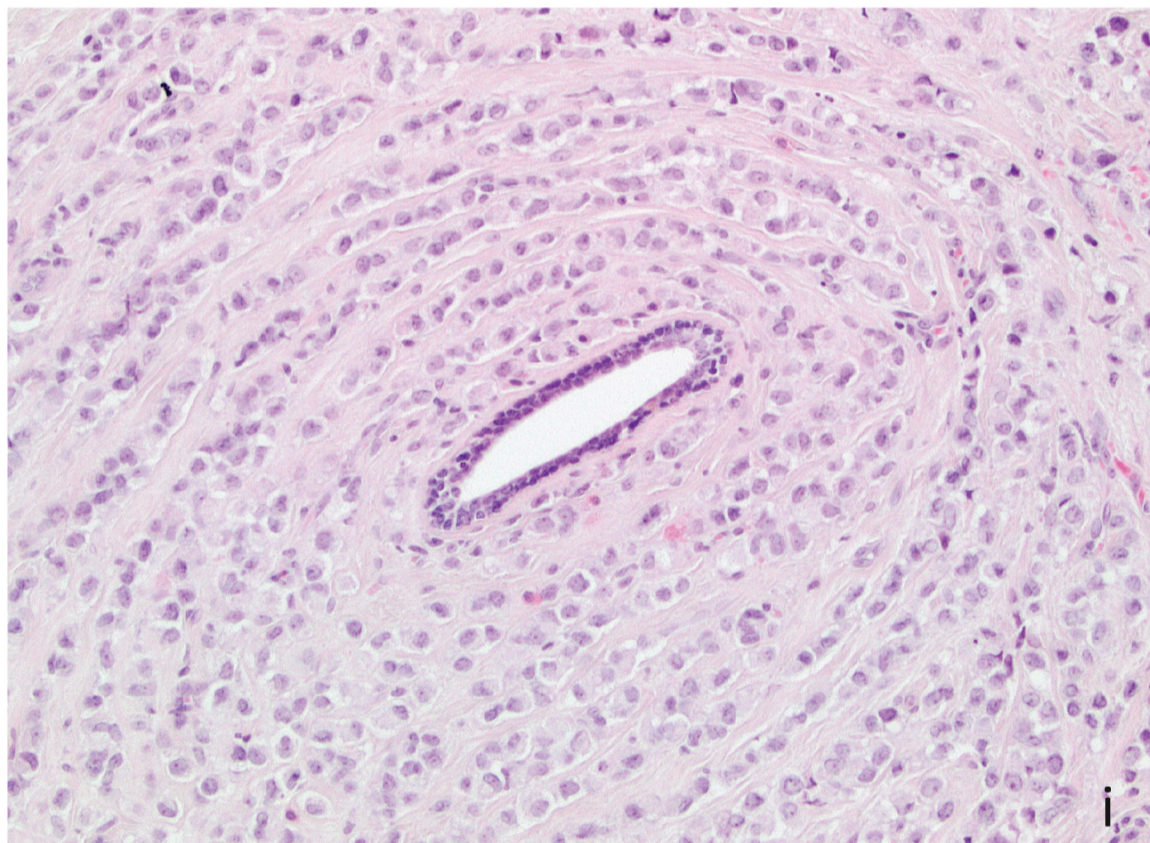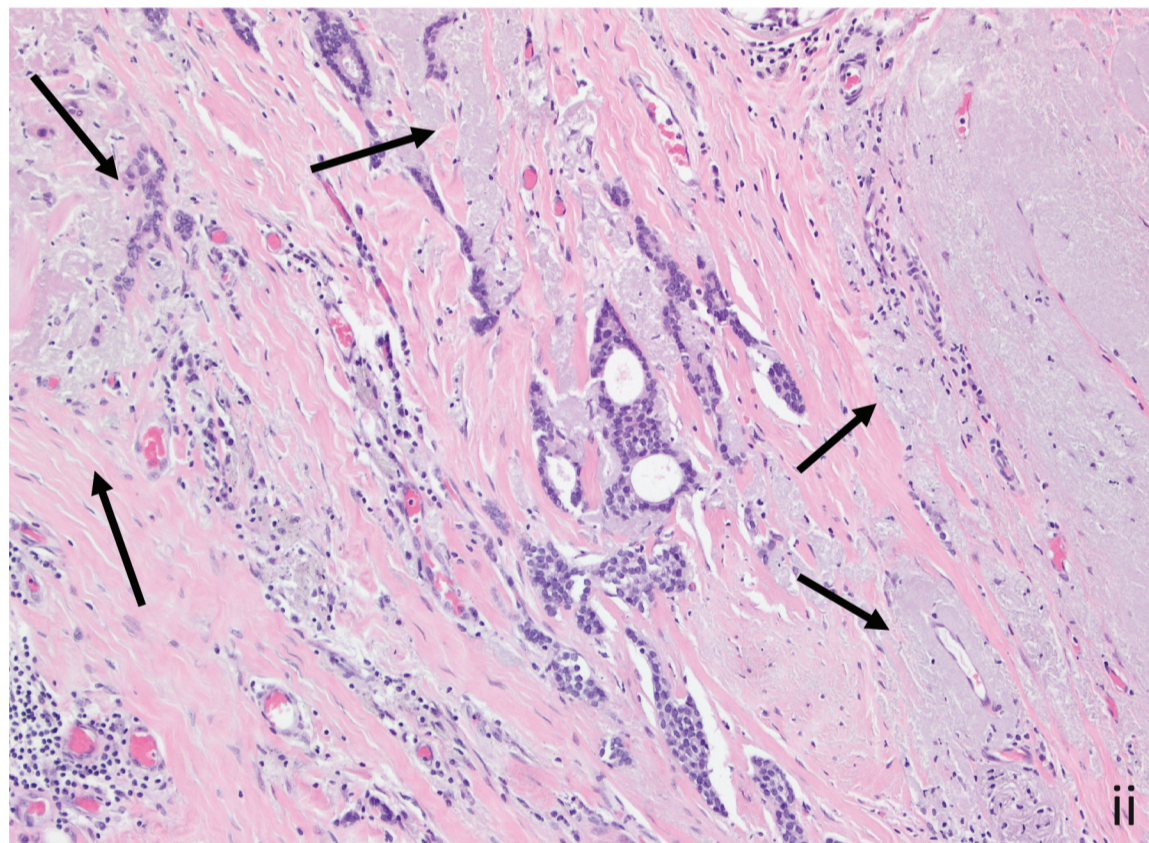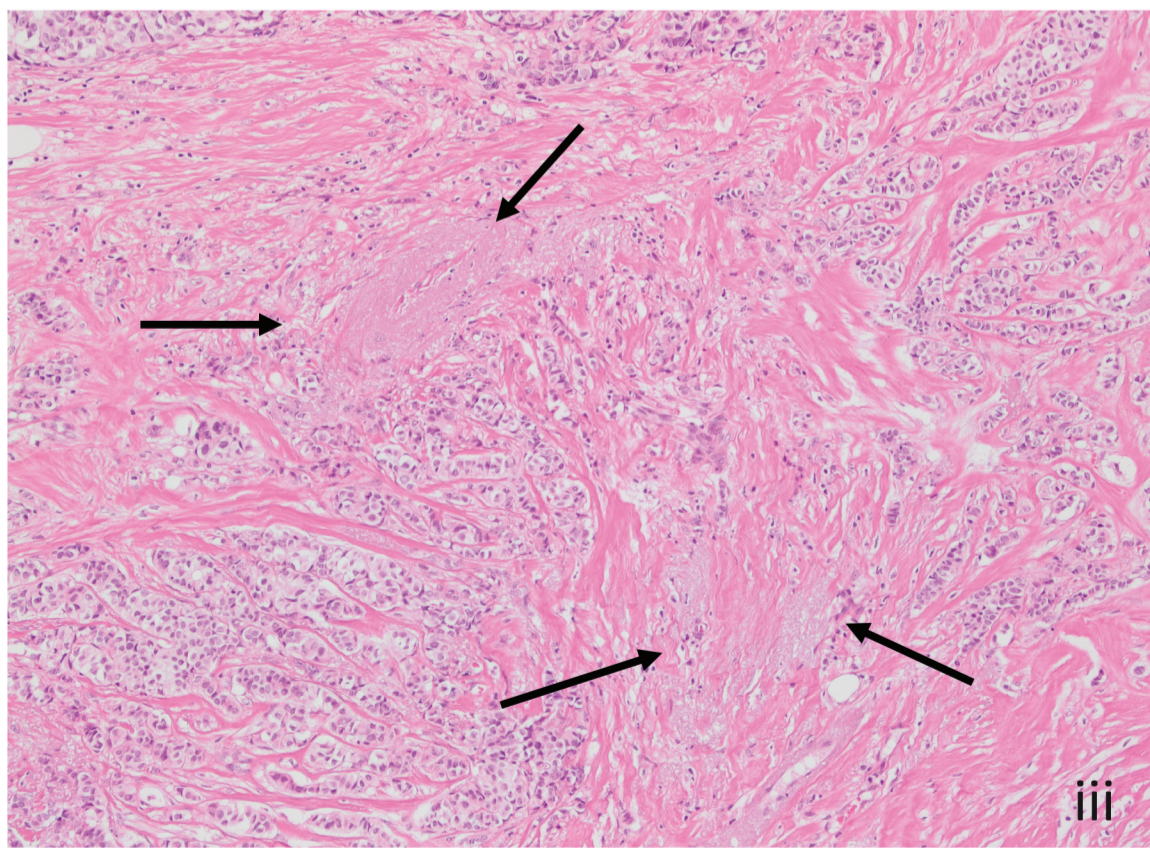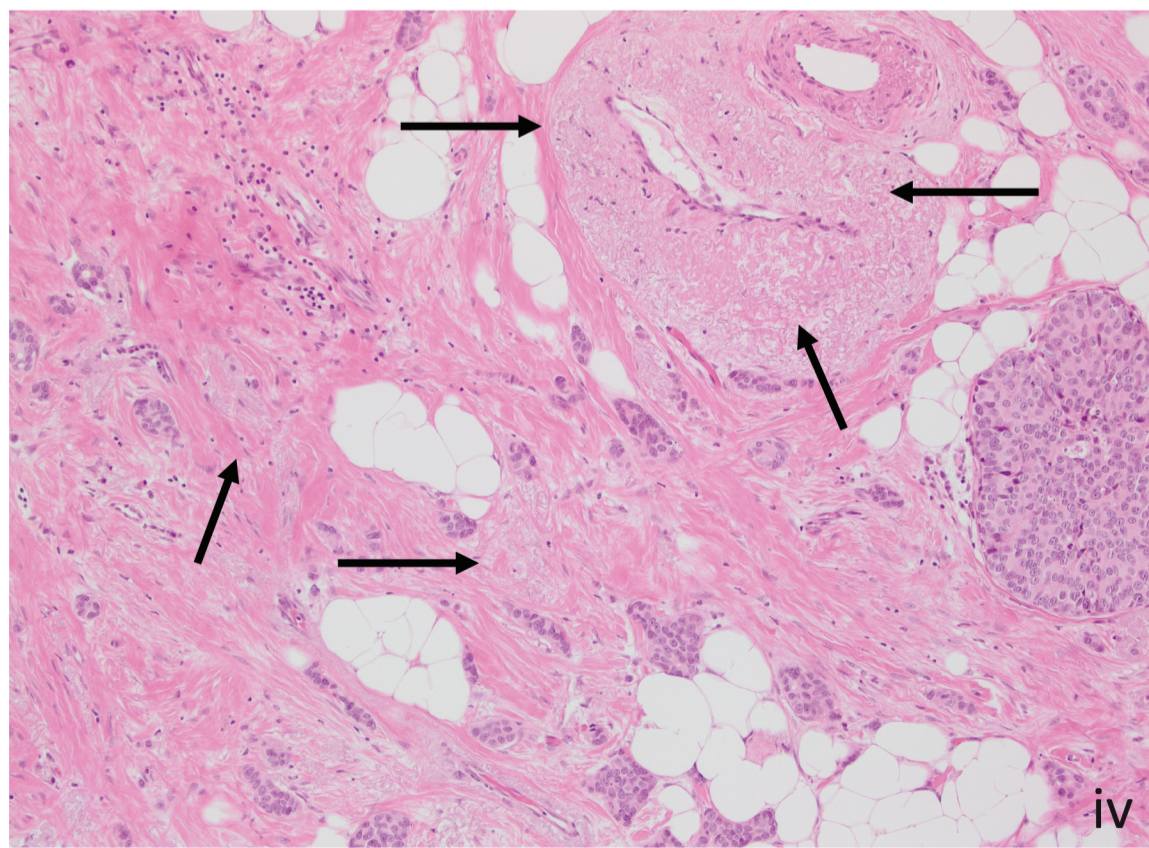

Fig.S7

Supplement: Supplementary file 4 — Additional file 4: Fig. S1. Mutational signatures of HLBCs. The picture reports the real 96-matrix substitution identified in the whole cohort and in each HER2 class. We estimated the mutational signatures comprising all the variants with a VAF>0.1. Donut and bar plots show the relative representation of the best fitting COSMIC v2 signatures with the substitutions identified in the cohorts. Fig. S2. A. Comparison of Variant Classification (VC) distribution among HLBCs and control cohorts. B. Comparison of Variant Type (VT) distribution among HLBCs and control cohorts. C. Comparison of variant pathogenic levels among HLBCs and control cohorts. Fig. S3. Hematoxylin and Eosin (H&E) and immunohistochemical reaction with antibodies raised against MLH1 for HLBC-1-17 characterized by a high TMB (42.52 mut/Mb), microsatellite instability, lack of MLH1 expression. Fig. S4. Heatmaps of pathway scores for HLBCs. A. Unsupervised clustering of pathways score. Annotation for IHC-based classes does not reveal enriched pathways associated with the IHC subclassification. B-C-D. Heatmaps representing the genes belonging to cytokine and chemokines signaling (B), antigen presentation (C), stromal markers (D). Some cases show peculiar upregulation (or downregulation) in these gene sets, regardless of the IHC class. Fig. S5. NMF basic features for the LAURA Classification. A. Line plot of the cophenetic coefficient determined by the rank summary. Coefficients tending to 1 indicate the most robust clusters. A large decrease in the stability was detected for a cluster with 6 groups. Red circled dots represent the selected grouping strategy, the first being the achievement of at least four groups without superimposable features with the PAM50 subtypes. B. Consensus matrix for the four clusters. Annotation for IHC-Class, IHC-subtype, PAM50-subtype, basic genes for the cluster definition, consensus group and NMF silhouette is provided. C. Heatmap reporting the genes driving the clustering. Fi [file 13073_2022_1104_MOESM4_ESM.pdf]
